# Slow and fast cortical cholinergic arousal is reduced in a mouse model of focal seizures with impaired consciousness

**DOI:** 10.1016/j.celrep.2024.115012

**Published:** 2024-12-05

**Authors:** Lim-Anna Sieu, Shobhit Singla, Jiayang Liu, Xinyuan Zheng, Abdelrahman Sharafeldin, Ganesh Chandrasekaran, Marcus Valcarce-Aspegren, Ava Niknahad, Ivory Fu, Natnael Doilicho, Abhijeet Gummadavelli, Cian McCafferty, Richard B. Crouse, Quentin Perrenoud, Marina R. Picciotto, Jessica A. Cardin, Hal Blumenfeld

**Affiliations:** 1 Department of Neurology, Yale University School of Medicine, New Haven, CT 06520, USA; 2 Department of Anatomy and Neuroscience Program, University College Cork, Cork, Ireland; 3 Department of Neuroscience, Yale University School of Medicine, New Haven, CT 06520, USA; 4 Department of Psychiatry, Yale University School of Medicine, New Haven, CT 06520, USA; 5 Department of Neurosurgery, Yale University School of Medicine, New Haven, CT 06520, USA; 6 Lead contact

## Abstract

Patients with focal temporal lobe seizures often experience loss of consciousness associated with cortical slow waves, like those in deep sleep. Previous work in rat models suggests that decreased subcortical arousal causes depressed cortical function during focal seizures. However, these studies were performed under light anesthesia, making it impossible to correlate conscious behavior with physiology. We show in an awake mouse model that electrically induced focal seizures in the hippocampus cause impaired behavioral responses to auditory stimuli, cortical slow waves, and reduced mean cortical high-frequency activity. Behavioral responses are related to cortical cholinergic release at two different timescales. Slow state-related decreases in acetylcholine correlate with overall impaired behavior during seizures. Fast phasic acetylcholine release is related to variable spared or impaired behavioral responses with each auditory stimulus. These findings establish a strong relationship between decreased cortical arousal and impaired consciousness in focal seizures, which may help guide future treatment.

## In brief

Sieu et al. report that impaired auditory responsiveness in a mouse temporal lobe seizure model is related to depressed cortical arousal and acetylcholine release. Slow changes in cortical slow waves, multiunit activity, and acetylcholine relate to impaired responsiveness in seizures, whereas fast changes relate to decreased responses to individual stimuli.

## Graphical abstract

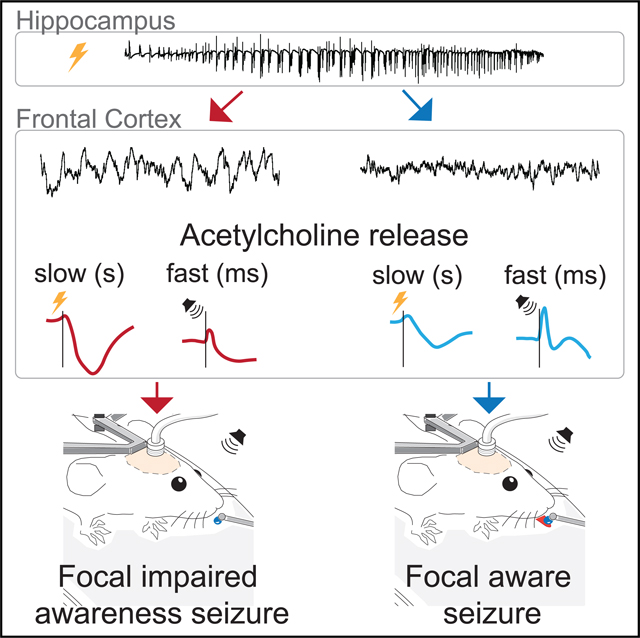

## INTRODUCTION

Temporal lobe epilepsy (TLE) is one of the most common and debilitating forms of focal epilepsy.^1,2^ TLE is characterized by seizures localized mostly to limbic structures, including the hippocampus. These seizures often cause functional deficits far beyond those expected from local impairment, including disabling loss of consciousness. Interestingly, patients with temporal lobe seizures and impaired consciousness show the expected high-frequency electrographic seizure activity in the temporal lobe, but slow oscillations (1–2 Hz) in frontal and parietal cortices, similar to a state resembling deep sleep, anesthesia, and coma.^3^ Human studies suggest that focal temporal lobe seizures lead to impaired consciousness by reducing subcortical arousal inputs causing a slow-wave, sleep-like state in the cortex, rather than by direct seizure propagation.^4,5^ The mechanism by which focal temporal lobe seizures−confined to a brain region devoted to memory, emotions, and related functions− can cause widespread depressed cortical function leading to loss of consciousness is not fully understood.

Previously studied rat models recapitulate key components of ictal unconsciousness in human TLE, including the transition from waking rhythms into slow-wave activity in the frontal cortex.^6–8^ Studies of rat cortical slow-wave activity observed in focal limbic seizures showed alternating up states of increased firing and down states of quiescence,^6,9^ similar to natural sleep or deep anesthetic conditions.^10–12^ Furthermore, cortical and thalamic cholinergic neurotransmission, critical for maintaining an arousal state,^13–15^ is reduced during seizures,^8^ notably attributable to a decrease of cholinergic neuronal firing activity from basal forebrain and the pedunculopontine tegmental nucleus.^8,16^ Moreover, other key regions of the subcortical arousal system, such as the intralaminar central lateral nucleus of the thalamus and the paratenial nucleus of the thalamus,^17,18^ showed reduced neuronal firing activity.^19,20^ Meanwhile, GABAergic systems known to be strongly connected to the hippocampus, such as the lateral septum (LS) and the anterior hypothalamus,^21,22^ showed increased blood-oxygen-level-dependent (BOLD) signals,^8^ suggesting an activation of inhibitory structures known to depress subcortical arousal systems. Further studies revealed that electrical stimulation of LS produced a transition to cortical slow-wave activity with reduced cortical cholinergic neurotransmission in the absence of seizures.^23^ Taken together, these findings strongly support the hypothesis that focal limbic seizures suppress subcortical arousal systems causing reduced cholinergic (and thus arousal) output to the cortex, resulting in impaired consciousness. This may occur via the activation of GABAergic systems such as the LS or anterior hypothalamus.

However, the above rat model studies were performed under light anesthesia, which can alter cortical and subcortical physiology, as well as the network effects of stimulation.^24^ Further, due to the anesthetized state, behavior, and therefore consciousness, was not directly assessed. We sought to investigate network, neurotransmitter, and neuronal mechanisms responsible for impaired consciousness during focal temporal lobe seizures in an awake and behaving animal model. Due to a broader availability of genetic and molecular tools and the ease of studying mice in an awake, head-fixed condition, we developed an awake, behaving head-fixed mouse model of TLE to measure the electrophysiological and behavioral effects of focal limbic seizures. This mouse model provides opportunities to identify neuronal mechanisms crucial for maintaining consciousness during focal limbic seizures and to explore the possibility of restoring cortical function and behavior,^25–28^ providing hope for treatment approaches to restore arousal in human epilepsy.^29^ This awake behaving mouse model of focal limbic seizures has been described recently by our group in preprint form^30^ and is presented here with additional experiments and results.

## RESULTS

### Unilateral electrical stimulation in dorsal hippocampus induces focal limbic seizures with reduced behavioral responses

**Figure 1.**
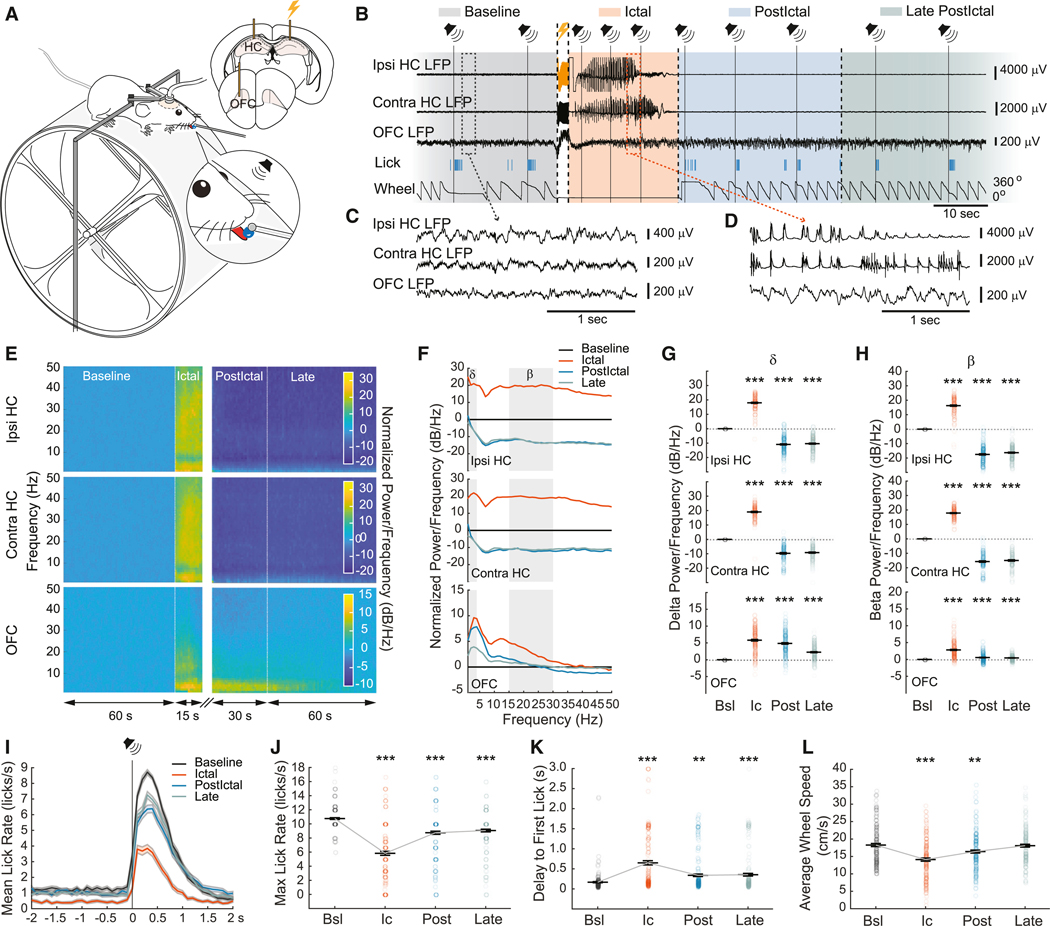
Induced focal limbic seizures in mice cause increased cortical slow waves and impaired behavioral responses (A) Diagrams illustrating the experimental apparatus and the recording setup. Mice were head-fixed and allowed to run on a running wheel while simultaneously obtaining recordings from the hippocampus (HC) and the orbitofrontal cortex (OFC). An auditory stimulus indicated water was available through a lick spout. Electrical stimulation to induce HC seizures is indicated by a lightning symbol. (B) Example traces from a single experiment. The auditory stimuli are represented by vertical lines and speaker symbols. Long vertical dashed lines surrounding the lightning symbol signify the start and end of the artifact from the electrical stimulus to HC. Shorter vertical dashed lines highlight the end or start of defined periods. Seizure activity is observed in the ipsilateral (Ipsi) and contralateral (Contra) HC following the unilateral electrical stimulation in the Ipsi HC (indicated by yellow artifact). The animal stopped licking to the auditory stimulus (as indicated by a pause in the vertical blue traces) and stopped running (indicated by no change in wheel position in bottom trace) during the ictal period. (C) Zoom-in of the LFP signals (gray box in B) showing representative baseline period. (D) Zoom-in of the LFP signals (red box in B) showing representative ictal period. Note that both HCs show seizure spikes, whereas OFC shows slow oscillation activity without seizure propagation. Note that the scale is 10 times smaller for Ipsi and Contra HC in (C) than in (D). (E) Time-frequency plots showing power change from average baseline power (normalized power) at defined periods: baseline, ictal, early postictal, and late postictal periods for Ipsi HC, Contra HC and OFC. Normalized power/frequency (dB/Hz) was calculated by normalizing power spectral density relative to baseline (see STAR Methods). (F) Power spectrum resulting from the time-frequency plots. Both HC signals showed large increase of power in a broad frequency range for both HCs signals, including the delta (δ) (1–4 Hz) and beta (β) (15–30 Hz) band power (gray boxes), during the ictal period, followed by an overall decrease of power after the end of seizure. The OFC signal showed maximum increase of power in the delta range and a smaller increase in the beta range during the ictal period, followed by a progressive decrease during the early and late postictal periods. (G and H) Scatterplots showing mean delta power (G) and mean beta power (H) changes during ictal, early postictal, and late postictal periods for Ipsi HC, Contra HC, and OFC. (I) Mean lick rates aligned to sound presentation (vertical line with speaker symbol) are compared at different periods. Ictal period showed a decreased lick rate compared to baseline. Note that the lick rate before the auditory stimuli is lower during the ictal period. (J and K) Scatterplot showing average of maximum lick rate (J) and average of delay to the first lick (K) following sound presentation during each period for each recording. Seizures significantly decrease lick rate (J) and increase latency to first lick (K). (L) Scatterplot showing average wheel speed for each period for recordings where mice were running (>10 cm/s) during the baseline period (*n* = 171 seizures, 26 animals). Mice showed significantly decreased wheel movement during seizures. For (E)–(K), *n* = 225 seizures in 26 animals. Data are shown as mean ± SEM. Significance was calculated by ANOVA with Bonferroni *post hoc* pairwise comparisons of baseline versus each of the other time periods. **p < 0.02 and ***p < 0.01. Bsl, baseline period; Ic, ictal period; Post, early postictal period; Late, late postictal period.

To produce a model of focal temporal lobe seizures, bipolar electrodes were implanted in the hippocampus (HC) to both induce and record seizures. An additional electrode was placed in the orbitofrontal cortex (OFC) to simultaneously record cortical activity (Figures S1 and S2) and to evaluate the presence of cortical slow waves during seizures. Performing a behavioral task requires a minimum of arousal and engagement; therefore as in humans, behavioral responsiveness was used as an indicator of level of consciousness^31–37^. In humans, answering basic questions is common practice to evaluate level of consciousness. As an alternative, mice performed an auditory-detection task. Water-restricted mice were head-fixed on a freely moving running wheel and trained to lick a drop of water from a dispenser located in front of their mouth in response to an auditory stimulus (Figure 1A). To determine if behavioral responses were affected by focal limbic seizures, seizures were induced by a unilateral, low-intensity electrical stimulus in the HC^6^ while mice were performing the auditory task (Figures 1A and 1B). HC and OFC local field potential (LFP) signals, sound presentations, lick responses, and wheel position were simultaneously recorded (Figure 1B). To obtain a focal seizure model, seizures with propagation of epileptiform activity into the OFC were excluded from analysis. We defined epileptiform seizure activity, as in prior human and animal recordings of HC seizures, as approximately 9- to 12-Hz repetitive spikes or polyspike and wave discharge.^6,38^ Unlike spontaneous human or animal model limbic seizures which show variable electrographic patterns,^39,40^ the morphology of seizures in our acute induced seizure model was relatively consistent. The ictal period was defined as the duration of the epileptiform seizure activity in the HC, observed after the electrical stimulation. The postictal period started right after the end of ictal period, divided into an early period fixed at a duration of 30 s, followed by a late postictal period of 60-s duration. The baseline period corresponded to the 60 s just prior to electrical stimulation. Seizures induced in the ipsilateral HC lasted between 1 and 40 s (8.5 ± 0.5 s, *n* = 225 seizures, 26 animals, mean ± SEM), while seizure propagation into the contralateral HC lasted between 5 and 40 s (13.9 ± 0.4 s, *n* = 156 seizures, 23 animals). In both the ipsilateral and contralateral HC, seizures showed an overall increase of power observed broadly across multiple frequency domains as compared to the baseline period, including delta (1–4 Hz) (ipsi HC, 17.98 ± 0.37 dB/Hz, *p* < 0.001; contra HC, 19.01 ± 0.40 dB/Hz, *p* < 0.001; mean ± SEM) and beta band (15–30 Hz) power (ipsi HC, 16.25 ± 0.43 dB/Hz, *p* < 0.001; contra HC, 17.84 ± 0.31 dB/Hz, *p* < 0.001) (Figures 1E–1H). No seizure propagation was recorded in OFC (*n* = 225 seizures, 26 animals) (Figures 1B–1D). Instead, similar to previously described rat studies,^6^ induced focal limbic seizures were associated with a large increase of delta power (5.81 ± 0.24 dB/Hz, *p* < 0.001, mean ± SEM) that persisted during the early postictal period (4.85 ± 0.22 dB/Hz, *p* < 0.001) and progressively decreased during the late postictal period (2.24 ± 0.15 dB/Hz, *p* < 0.001) (Figures 1E–1G). Other frequency bands showed smaller increases of power (Figures 1E–1H).

To investigate the effect of focal limbic seizures on behavior relevant to arousal, we examined lick responses to an auditory stimulus and measured running by wheel speed during each session. The average duration of a session was 18.8 ± 0.6 min (mean ± SEM) before mice became satiated to the water reward and stopped responding. During the baseline period, mice responded to the auditory stimulus by licking 100% (730 out of 730 stimuli, *n* = 225 seizures) of the time, with a maximum lick rate of 10.76 ± 0.12 licks per second (mean ± SEM) (Figures 1I and 1J). The first lick response occurred on average 0.17 ± 0.01 s after the sound (mean ± SEM) (Figure 1K). Compared to baseline, induced focal limbic seizures significantly reduced responsiveness to auditory stimuli during the ictal period (maximum lick rate, 5.83 ± 0.28 licks/s, *p* < 0.001; delay, 0.65 ± 0.05 s, *p* < 0.001; mean ± SEM), early postictal period (maximum lick rate, 8.74 ± 0.22 licks/s, *p* < 0.001; delay, 0.34 ± 0.03 s, *p* < 0.01), and late post-ictal period (maximum lick rate, 9.06 ± 0.19 lick/s, *p* < 0.001; delay, 0.35 ± 0.03 s, *p* < 0.01) with return to normal responses later (Figures 1I–1K). Spontaneous licks that occurred within a period of 2 s before the auditory stimulus, reflecting anticipation, were also significantly reduced during the ictal period compared to baseline and postictal periods (ictal, 0.44 ± 0.08 licks/s, baseline, 1.24 ± 0.12 licks/s, *p* < 0.001; early postictal, 1.04 ± 0.13 licks/s, *p* < 0.001; late postictal, 0.97 ± 0.10 licks/s, *p* < 0.001; mean ± SEM) (Figure 1I). Sham stimulations did not evoke any significant difference in behavioral responses compared to baseline period (Figures S3C–S3E). Additionally, analysis of wheel speed showed that mice run significantly less during the ictal period (baseline, 18.26 ± 0.44 cm/s; ictal, 14.10 ± 0.45 cm/s, *p* < 0.001; mean ± SEM; *n* = 171 seizures) and early postictal period (16.41 ± 0.43 cm/s, *p* = 0.0145; *n* = 171 seizures) compared to baseline (Figure 1L), while no change was seen during sham stimulation (*n* = 63 sessions) (Figure S3F), further supporting the concept that behavioral arrest during seizures in mice is similar to human temporal lobe seizures.^41,42^ Interestingly, while licking behavior overall decreased during seizures, the responses were highly variable, and ranged from being impaired to normal during the ictal period (Figures 1J and 1K), suggesting that consciousness was not always impaired during seizures, an observation similarly seen in humans.^5,43^

### Focal limbic seizures depress cortical function

**Figure 2.**
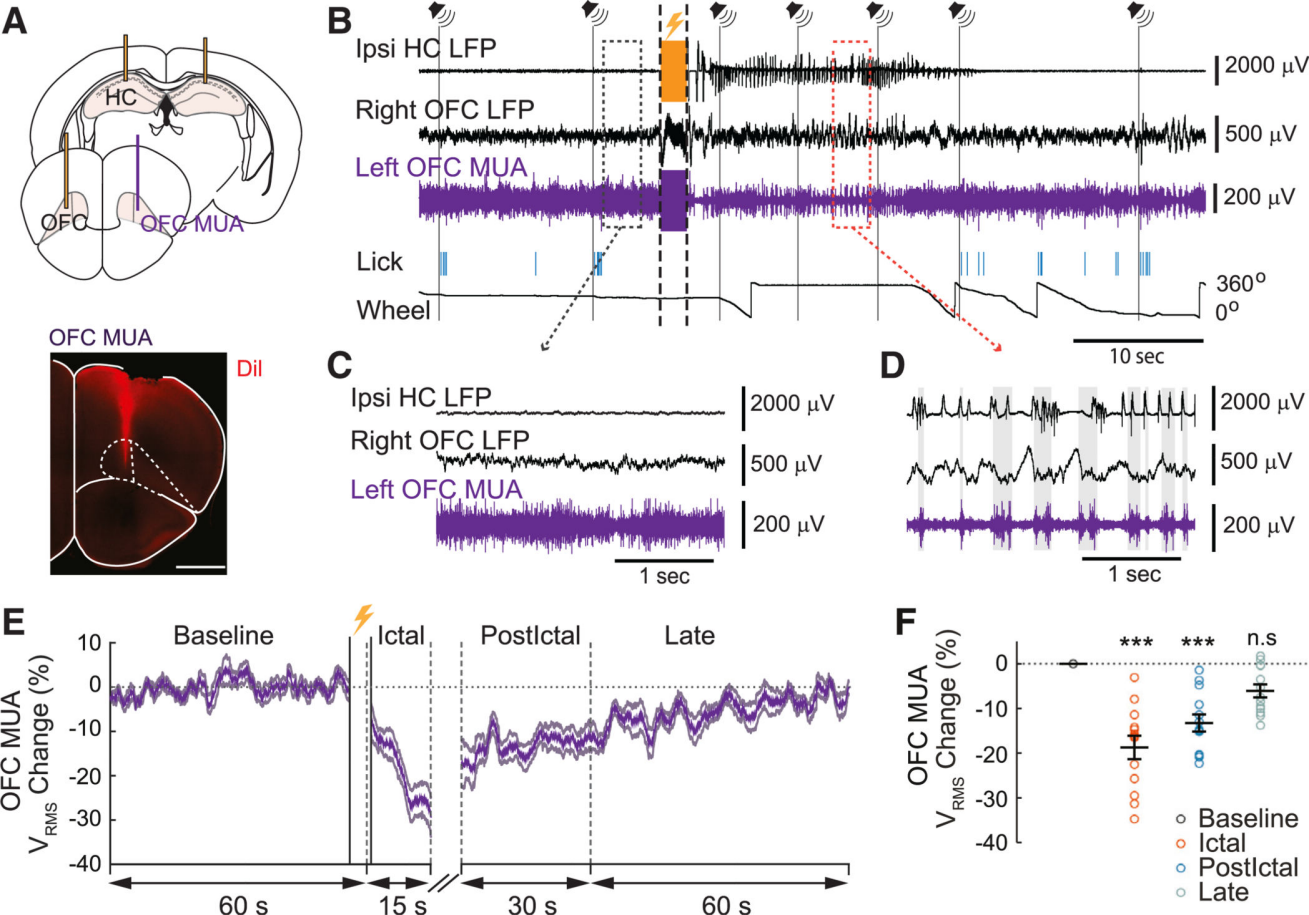
Decreased activity of cortical neurons during focal limbic seizures resembles slow-wave sleep A) Schematic drawing showing general location of the unipolar multiunit activity (MUA, purple) electrode in OFC added among the bipolar local field potential (LFP) electrodes (yellow) and an example of an MUA electrode location marked by the fluorescent 1,1′’-dioctadecyl-3,3,3′’,3′’-tetramethylindocarbocyanine Perchlorate (DiI) agent in OFC after performing histology in one animal. Scale bar: 1 mm. (B) Example of a recording session including the MUA signal (purple) from the OFC. Symbols and marking of events during the recording follow the same conventions as in Figure 1. (C) Zoom-in of the tonic activity of the OFC neuronal population recorded from the MUA electrode during baseline from the gray box in (B). (D) Zoom-in of the alternating up- and down-state activity during the seizure from the red box in (B). Note that neuronal activity (indicated by gray shading) matched the OFC LFP up-state oscillations rather than HC seizure spike activity. (E) Mean time courses of MUA V_RMS_ change from baseline, aligned at the start of seizure, showed a decrease of neuronal population activity during the ictal period that progressively increased back to baseline during the early postictal and late postictal periods. The two full vertical black lines before the start of the ictal period in (E) correspond to a cut artifact period caused by the electrostimulation. (F) Scatterplot showing significant decrease of mean MUA V_RMS_ change during the ictal and early postictal periods compared to the baseline period. For (E) and (F), *n* = 13 seizures in 10 animals. Data are indicated as mean ± SEM. Significance was calculated with ANOVA with Bonferroni *post hoc* pairwise comparisons of baseline to each of the other periods. ***p < 0.001 and n.s, non-significant.

Slow-wave activity that was found in the OFC during focal limbic seizures in mice resembles slow-wave sleep. However, it is possible that these cortical changes were directly driven by seizure propagation instead of representing a distinct state of depressed cerebral function. To determine if cortical changes more closely resemble seizure activity or sleep, we recorded multiunit activity (MUA) in the left OFC in 11 mice (Figures 2A and S1B). We found that MUA was markedly reduced during the ictal period, compared to baseline (Figure 2B). In addition, MUA tended to alternate between an up- and down-state pattern of neuronal activity during ictal slow-wave activity (Figure 2D), in contrast to the baseline tonic activity pattern (Figure 2C). MUA amplitude (V_RMS_) was used as surrogate marker of neuronal firing as in prior work^6,19,44,45^ (Figure S4). In agreement with previous work investigating cortical slow-wave oscillations,^6–9^ we found that, during the ictal period, the fluctuations in cortical MUA V_RMS_ were more often correlated with the cortical slow waves than with HC seizure activity (Figure S5; Table S1). This suggests that cortical slow waves are not directly driven by seizure activity but rather correspond to a depressed arousal state resembling sleep. On average, MUA amplitude was significantly reduced during the ictal period (−19% ± 0.3% V_RMS_ change, *p* < 0.001; mean ± SEM, *n* = 13 seizures) and the early postictal period (−13% ± 0.2% V_RMS_ change, *p* < 0.001) (Figures 2E and 2F), while it gradually recovered in the late postictal period (−6% ± 0.1% V_RMS_ change, *p* = 0.12). These findings suggest that slow waves found in OFC during focal limbic seizures represent a distinct state of depressed cerebral function more closely resembling sleep than seizures.

### Cortical slow waves during seizures are associated with impaired behavioral responses

In patients, a transition from waking rhythms to slow-wave oscillations (1–2 Hz) is observed in frontal and parietal association cortex during temporal lobe seizures with impaired consciousness (focal impaired awareness seizures), whereas seizures without loss of consciousness (focal aware seizures) do not show this change.^4,5^ As was already mentioned, like human TLE, our mouse model replicated the variability of behavioral impairment during seizures, with some seizures resulting in impaired and others in normal behavioral responses (Figures 1J–1L). We sought to determine if the model also reproduced the association between more severe cortical slow-wave activity and behavioral impairment during seizures.

To correlate OFC LFP activity to behavior, we classified behavioral responses into two categories, “hit” for spared response, reflecting spared consciousness, and “miss” corresponding to impaired response, reflecting impaired consciousness, based on latency to the first lick in response to the auditory stimulus. A hit was defined as a first lick that occurred within a 1-s window after the auditory stimulus, and a miss as no lick, or a first lick that occurred after the 1-s window following the auditory stimulus. We then classified seizures into three types based on behavioral responses during the ictal period: “spared seizure,” only hits to auditory stimuli (*n* = 72 out of 225 seizures, 16 out of 26 animals); “impaired seizure,” only misses (*n* = 37 out of 225 seizures, 13 out of 26 animals); and “mixed seizure,” both hits and misses (*n* = 116 out of 225 seizures, 25 out of 26 animals).

**Figure 3.**
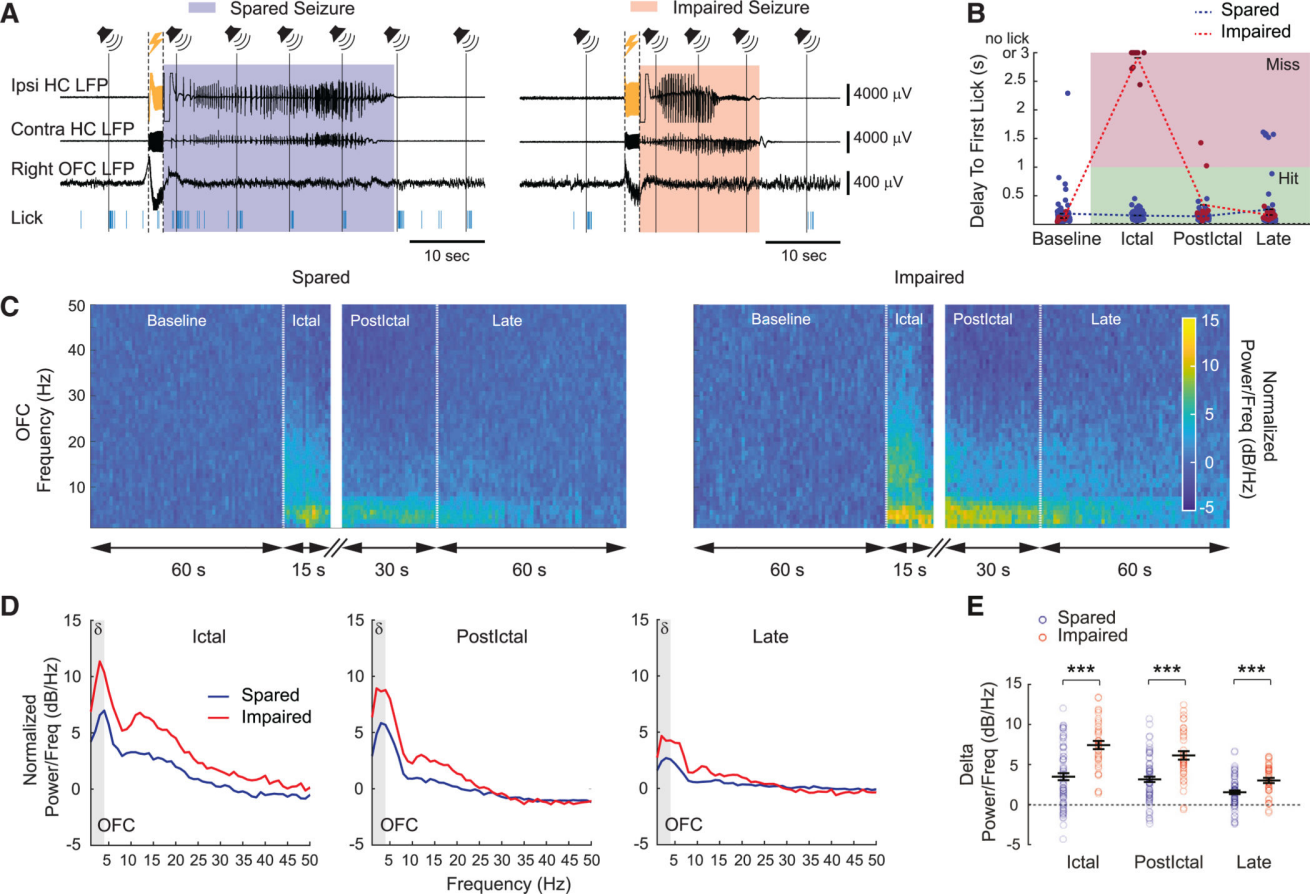
Impaired seizures are associated with more cortical slow waves (A) Example of a spared and an impaired seizure defined by the presence or absence of licking responses within a 1-s window after the auditory stimuli during the ictal period. Symbols and marking of events during the recording follow the same conventions as in Figure 1. (B) Graph showing details on spared and impaired seizures classification. A licking response is considered as a “hit” if the first lick occurred within a 1-s window after the auditory stimulus (green region), while it becomes a “miss” if the first lick happened after the 1-s window following sound or if there is no lick (pink region). A seizure is defined as “spared” if every licking responses during the ictal period is a hit (blue symbols) or is defined as “impaired” if every response during the ictal period is a miss (red symbols). (C and D) Time-frequency plots (C) and power spectra (D) showing normalized power change of OFC LFP at different periods during spared seizures versus impaired seizures. Normalized power/frequency (dB/Hz) was calculated by normalizing power spectral density relative to baseline (see STAR Methods). Note a higher increase of delta power (*δ*) (1–4 Hz, gray regions in D) during the impaired seizures. (E) Comparison of mean delta power changes between spared and impaired seizures during ictal, early postictal, and late postictal periods. For (B)–(E), spared, *n* = 72 seizures in 16 animals; impaired, *n* = 37 seizures in 13 animals; 23 animals in total. Data are indicated as mean ± SEM. Significance was calculated with Mann-Whitney U test for spared versus impaired seizures in each period. ***p < 0.001.

We examined OFC LFP activity for spared and impaired seizures (Figures 3A and 3B). Power spectra were calculated for OFC LFP signals for each period, with the ictal period truncated at 15 s (Figure 3C). OFC LFP signals showed higher power in the delta band for impaired seizures compared to spared seizures during the ictal period (7.43 ± 0.51 dB/Hz and 3.48 ± 0.44 dB/Hz, respectively, *p* < 0.001; mean ± SEM), early postictal period (6.13 ± 0.53 dB/Hz and 3.15 ± 0.33 dB/Hz, respectively, *p* < 0.001), and late postictal period (3.01 ± 0.32 dB/Hz and 1.53 ± 0.23 dB/Hz, respectively, *p* < 0.001) (Figures 3C–3E). This indicates that seizures with impaired behavioral responses have significantly more cortical slow-wave activity than spared seizures. Furthermore, we found that impaired seizures exhibited greater propagation of fast ictal activity to the contralateral hippocampus (Figure S6), demonstrating similarities to human TLE.^5^

**Figure 4.**
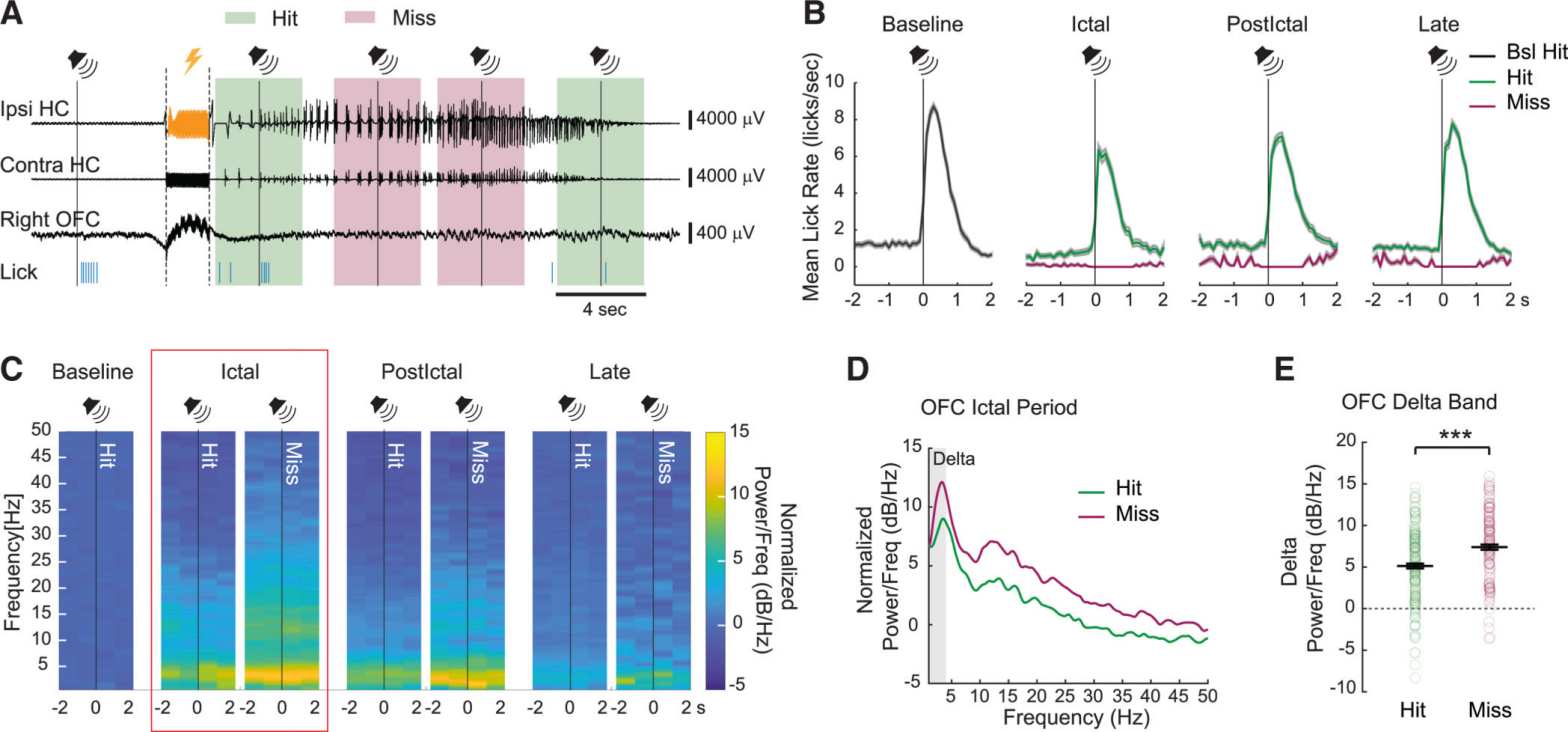
Miss responses during seizures are accompanied by more cortical slow waves than hit responses (A) Example of a recording with a seizure composed of both hit (green background) and miss (pink background) responses to the auditory stimuli during the ictal period. Symbols and marking of events during the recording follow the same conventions as in Figure 1. (B) Comparison of mean lick rates aligned to sound presentation (vertical line with speaker) at different periods for hit versus miss responses. All miss responses showed no licking responses within a 1-s window after the auditory stimuli; in hit responses, the first lick did occur within 1 s. Note that the baseline lick rate before the auditory stimuli was lower during the miss responses compared to the hit responses (see text). (C) Time-frequency plots showing normalized power change of OFC LFP in ±2-s data segments around the time of auditory stimuli (vertical line with speaker) for the hit and miss responses at different periods. Normalized power/frequency (dB/Hz) was calculated by normalizing power spectral density relative to baseline (see STAR Methods). Miss responses showed higher increase in delta power (versus baseline hit period) compared to the hit responses. (D) Comparison of the power spectrum of the ictal OFC LFP from the hit and miss responses (red box in C) showing a higher increase of delta power (gray shading, 1–4 Hz) during the miss responses than the hit. (E) Mean delta power changes of the ictal OFC LFP showing significant difference between hit versus miss responses (same data segments as C red box, and D). For (B)–(E), ictal, *n* = 166 hits and *n* = 140 misses; early postictal, *n* = 216 hits and *n* = 51 misses; late postictal, *n* = 220 hits and *n* = 51 misses during 225 seizures in 26 animals. Data are indicated as mean ± SEM Significance was calculated with Mann-Whitney U test. ***p < 0.001.

To confirm the correlation between cortical slow waves and behavior, we focused our analysis on signals recorded within a time windows of 2 s before and 2 s after each sound presentation (Figure 4A). OFC LFP activity was compared for all hit and miss responses, for all seizures. As already described, miss responses were defined as no licks within a 1-s window after the auditory stimulus, and hit responses had licks within the 1-s window after the auditory stimulus. Note that a higher baseline spontaneous lick rate was observed before the auditory stimuli for the hit responses compared to the miss responses during ictal (hit, 0.66 ± 0.12 licks/s, *n* = 166 sounds; miss, 0.09 ± 0.04 licks/s, *n* = 140 sounds; *p* < 0.001; mean ± SEM), early postictal (hit, 1.17 ± 0.14 licks/s, *n* = 216 sounds; miss, 0.23 ± 0.07 licks/s, *n* = 51 sounds; *p* < 0.001), and late postictal periods (hit, 1.02 ± 0.11 licks/s, *n* = 220 sounds; miss, 0.26 ± 0.09 licks/s, *n* = 51 sounds; *p* < 0.001) (Figure 4B), suggesting that animals were in a different behavioral arousal state around the time of miss versus hit responses. To investigate this further, a power spectrum was calculated for OFC LFP signals within the time windows of 2 s before and 2 s after each classified hit or miss sound stimulus. We found that there was a significant increase in cortical delta band power around the time of both hit and miss responses during seizures; however, the miss responses had a significantly higher change compared to the hit responses (hit, 5.12 ± 0.29 dB/Hz, *n* = 166 sounds; and miss, 7.39 ± 0.32 dB/Hz, *n* = 140 sounds; *p* < 0.001; mean ± SEM) (Figures 4C–4E), confirming that impaired responses are correlated with stronger cortical slow waves during the ictal period. A similar result was observed during the early postictal period with miss responses showing higher delta power compared to hit responses (hit, 4.77 ± 0.28 dB/Hz, *n* = 216 sounds; miss, 6.35 ± 0.38 dB/Hz, *n* = 51 sounds; *p* = 0.017), whereas the late postictal period did not show any significant difference between hit and miss (hit, 1.97 ± 0.19 dB/Hz, *n* = 220 sounds; miss, 1.82 ± 0.35 dB/Hz, *n* = 51 sounds, *p* = 0.83). These results indicate that impaired behavior in our mouse seizure model is associated with increased cortical slow waves, suggesting that cortical arousal is depressed.

### Decreased slow and fast cortical acetylcholine release related to impaired behavior during seizures

**Figure 5.**
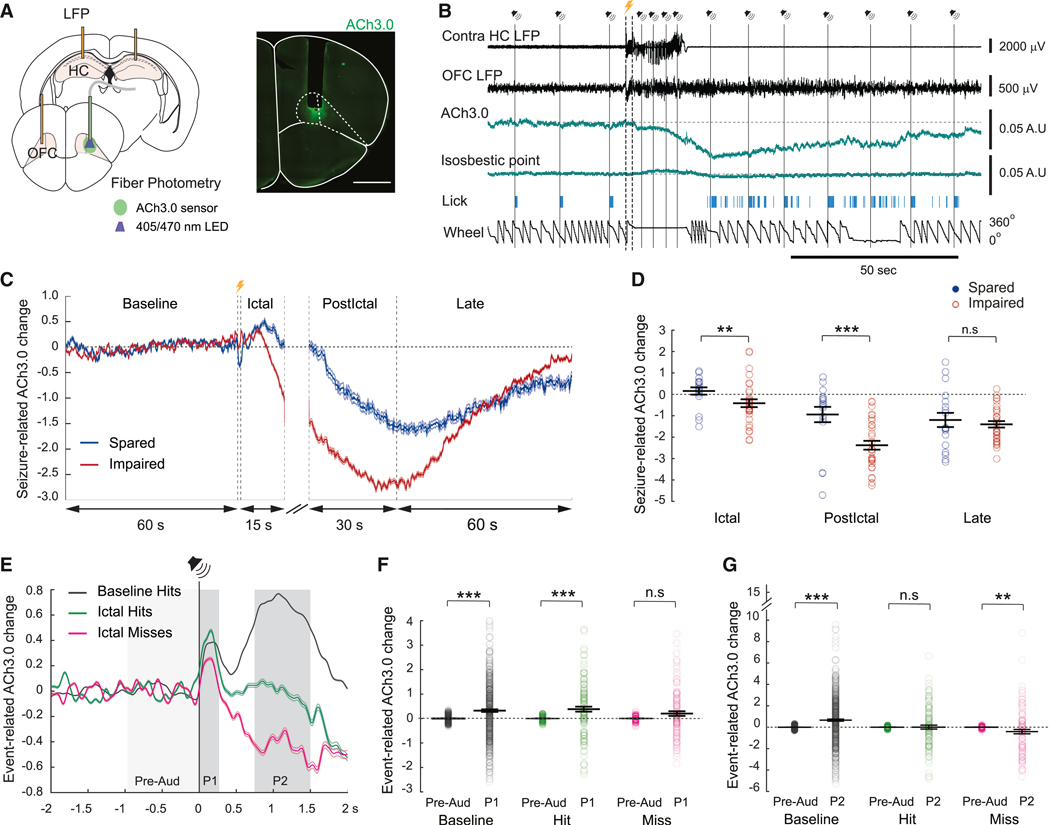
Cortical ACh neurotransmission is reduced during focal limbic seizures in relation to behavior (A) Schematic drawing showing general location of the implanted optic fiber (blue/green line) for fiber photometry recording, added among the LFP electrodes (in yellow) and an example of histology showing the tract of the optic fiber located above the region of GFP fluorescence from expression of the ACh3.0 sensor in OFC. Scale bar: 1 mm. (B) Example of a recording session including the ACh3.0 GFP signal and the isosbestic point (reference) signal. ACh3.0 signal decreased during the seizure induced by HC electrical stimulation (lightning symbol surrounding by dashed lines), while the isosbestic point signal did not show major changes. Note that the animal stopped licking (vertical blue traces, in response to sound stimuli indicated by speaker symbols and vertical lines) and running (no change in wheel position) during the seizure. (C) Mean time courses of seizure-related ACh3.0 change from behaviorally spared versus impaired seizure recordings, aligned at the start of seizure (ictal), then at start of early postictal period (postictal), and continuing into the late postictal period (late). ACh3.0 signal showed larger decrease during impaired seizures versus spared seizures. (D) Mean seizure-related ACh3.0 changes showing significantly larger reduction during the ictal and early postictal periods from impaired seizures compared to spared seizures. (E) Mean event-related ACh3.0 changes aligned to sound presentation (vertical trace with speaker) are compared between the baseline hit, ictal hit, and ictal miss responses. Two fast ACh evoked response phases are seen: a first release phase (P1, dark gray box from 0 to 250 ms) and a second release phase (P2, dark gray box from 750 ms to 1.5 s). A pre-auditory stimuli baseline period was defined from −1 to 0 s (Pre-Aud, light gray box). (F) Comparison of mean event-related ACh3.0 changes between the pre-auditory stimuli period (Pre-Aud) and the first ACh release phase (P1) for the baseline hit, ictal hit, and ictal miss responses showing significant increases of P1 versus Pre-Aud for the baseline and ictal hit responses, whereas there is no significant increase for the ictal miss responses. (G) Comparison of mean event-related ACh3.0 changes between the pre-auditory stimuli period (Pre-Aud) and the second ACh release phase (P2) for the baseline hit, ictal hit, and ictal miss responses showing significant increase of P2 versus Pre-Aud for the baseline hit responses, no significant difference for the ictal hit responses, and a significant decrease of P2 versus Pre-Aud for the ictal miss responses. For (C) and (D), spared, *n* = 18 seizures; impaired, *n* = 31 seizures in seven animals. For (E–G), *n* = 607 baseline hits, 128 ictal hits, 119 ictal misses during 118 seizures in seven animals. Data are indicated as mean ± SEM Significance was calculated with Mann-Whitney U test. **p < 0.02; ***p < 0.01; and n.s, not significant.

Because cholinergic neurotransmission into neocortex is known to mediate arousal,^13–15^ we hypothesized that acetylcholine release will be reduced in OFC during impaired awareness seizures in contrast to spared aware seizures. To test this hypothesis, we measured cortical acetylcholine (ACh) levels using a genetically encoded GPCR-activation-based (GRAB) ACh sensor that converts an ACh-induced conformational change on a modified type 3 muscarinic receptor into a sensitive green fluorescent protein (GFP) optical response (ACh3.0).^46,47^ We expressed the sensor in the left OFC via a local injection of adeno-associated virus encoding the sensor and implanted an optical fiber to record ACh signaling (Figure 5A). A typical example of a behaviorally impaired seizure is shown in Figure 5B, where there is a decrease in OFC fluorescence during the ictal period (indicating a decrease in ACh levels) that persisted during the early postictal period, and then gradually returned toward baseline during the late postictal period. The isosbestic point of fluorescence, simultaneously measured as a reference, showed minimal changes during the seizure (Figure 5B). On average, seizures caused a non-significant increase of ACh3.0 signal that lasted for a few seconds following stimulation before a decrease was observed during the ictal period (Figure 5C). The ACh3.0 reduction continued during the early postictal period and then gradually returned toward baseline levels during the late postictal period. Control sham stimulations without seizure were also conducted and showed no significant ACh3.0 signal change during control and post-control periods compared to baseline (Figures S7A and S7B). Comparison between ACh3.0 change during spared seizures (*n* = 18) and impaired seizures (*n* = 31) revealed significantly more decreased fluorescence for impaired seizures during the ictal period (spared, 0.16 ± 0.17 mean ACh3.0 change, impaired, −0.4 ± 0.19 mean ACh3.0 change, *p* = 0.011; mean ± SEM) and the early postictal period (spared, −0.94 ± 0.36; impaired, −2.38 ± 0.20 mean ACh3.0 change, *p* < 0.001), but no significant difference during the late postictal (spared, −1.20 ± 0.33; impaired, −1.40 ± 0.15 mean ACh3.0 change, *p* = 0.73) (Figure 5D). These findings suggest that a greater decrease of cortical ACh levels during seizure activity and extending into the postictal period is related to more severely impaired ictal behavioral responses. Cortical ACh level is known to be low during slow-wave sleep. Accordingly, during anesthesia, ACh3.0 measurement showed an overall lower level of signal compared to the awake state (Figures S8B and S8C). Electrical stimulation of the basal forebrain, an area known to contain cholinergic neurons that project into neocortex,^48–50^ transiently increased OFC ACh3.0 signal during anesthesia to briefly reach the level of the awake state in the example shown in Figures S8A–S8C. These findings support the idea that measurements of decreased ACh3.0 signals during seizures reflect depressed cortical ACh release comparable to other decreased arousal states such as anesthesia.

Interestingly, evoked ACh release with shorter timescales can occur in medial frontal cortex in response to rewarding cues.^51–53^ We therefore examined fast evoked ACh3.0 fluorescence changes in OFC in response to reward-paired sound presentations to determine if transient ictal hit and miss ACh responses may differ from transient ACh responses during preictal baseline. Averaging ACh3.0 fluorescence changes from auditory hit responses during the baseline period showed two distinct fast positive transient phases: a first phase defined from 0 to 250 ms (P1) and a second phase defined from 750 to 1500 ms (P2) after sound stimuli (Figure 5E). We compared the P1 and P2 ACh release phases to a pre-auditory baseline, defined by the mean ACh3.0 signal at −1,000 to 0 ms before sound presentations for baseline hit, ictal hit, and ictal miss responses. We found significant fast evoked ACh3.0 transient signals during P1 for baseline hit responses versus pre-auditory signals (Pre-Aud, 0.0032 ± 0.0054; P1, 0.32 ± 0.05 mean ACh3.0 change, *n* = 607 sounds, *p* < 0.001; mean ± SEM) and for ictal hit responses (Pre-Aud, 0.0038 ± 0.0093; P1, 0.38 ± 0.1 mean ACh3.0 change, *n* = 128 sounds, *p* < 0.001) but a non-significant evoked signal for ictal miss responses (Pre-Aud, 0.0039 ± 0.0104; P1, 0.20 ± 0.09 mean ACh3.0 change, *n* = 119 sounds) (Figures 5E and 5F). These data suggest that the evoked ACh release, in response to previously rewarded sound presentations, is disrupted during impaired (miss) responses. During the second release phase (P2), ACh3.0 signals increased significantly for the baseline hit responses compared to pre-auditory baseline (P2, 0.66 ± 0.10 mean ACh3.0 change, *n* = 128 sounds, *p* < 0.001; mean ± SEM) (Figure 5G). The ictal miss P2 phase was significantly decreased when compared to pre-auditory baseline (P2, −0.41 ± 0.20 mean ACh3.0 change, *n* = 119 sounds, *p* = 0.017) reflecting the continued longer-term ictal decrease in ACh already described (e.g., Figure 5C). The decrease during P2 for ictal hits was less prominent and not significantly different from baseline (P2, 0.0091 ± 0.18 mean ACh3.0 change). We hypothesized that the P2 phase of ACh signals arising 750–1,500 ms after auditory stimuli may arise from increased cholinergic arousal related to licking. To determine if the P2 release phase was related to the licking response, we analyzed the ACh3.0 signals in response to sound presentations during the period where mice were satiated and therefore no longer licked in response to sounds in sessions without seizure induction. On average, a significant positive ACh3.0 transient P1 phase signal was observed (Pre-Aud, −0.03 ± 0.02; P1, 0.47 ± 0.15 mean ACh3.0 change, *n* = 90 sounds, *p* = 0.001; mean ± SEM), whereas no significant change was observed in the P2 phase in the absence of licking (P2, −0.05 ± 0.22 mean ACh3.0 change, *p* = 0.93) (Figure S9), suggesting that the evoked P2 phase is associated with the licking response, whereas ACh release in P1 may represent transient arousal in response to reward-associated cue perception.

## DISCUSSION

We developed an awake mouse model of focal limbic seizures with similar key characteristics to those observed in human temporal lobe seizures. In particular, the mice exhibited cortical sleep-like slow waves associated with impaired behavioral responsiveness. We established that the cortical slow waves exhibited up and down states in neuronal activity, producing an overall decrease of mean cortical neuronal activity consistent with depressed arousal. Using a genetically encoded fluorescent ACh sensor, we found depressed cortical cholinergic arousal on several timescales related to impaired behavior. Slow decreases in ACh neurotransmission during focal limbic seizures and extending well into the postictal period were associated with overall impaired behavioral responses during seizures. In addition, transient fast increases in ACh were observed during the auditory task, including a first phase likely related to auditory cue perception and a second phase associated with the behavioral licking response. Dynamic moment-to-moment variability in behavioral responses during seizures was related to these faster components of arousal modulation. These findings provide a conceptual framework for understanding the effects of impaired arousal on behavioral responsiveness and conscious experience.

Our mouse model replicated focal impaired and focal aware seizures similar to those seen in human TLE, where cortical slow-wave activity during seizures is associated with impaired behavioral responsiveness.^4,5^ This model also has important advantages for investigating fundamental mechanisms of arousal changes during focal limbic seizures. We found that that there was a decrease in OFC cortical high-frequency V_RMS_, a surrogate marker of neuronal firing^6,19,44,45^ during focal limbic seizures in the mouse model, with up and down states resembling slow-wave sleep, encephalopathy or deep anesthesia^10–12^ similar to previous work in lightly anesthetized rats.^6,9^ Our study goes several important steps further by providing direct neuronal recordings in an awake behaving seizure model showing altered cortical physiology and decreased cortical high-frequency signals related to neuronal firing.

Additionally, we show here that focal limbic seizures are associated with a decrease of cortical cholinergic input, a feature that has only been observed in the rat model under light anesthesia.^8^ Due to our awake, behaving head-fixed model, we were able to further show that focal limbic seizures with impaired behavior had a larger decrease of cortical cholinergic input than those with spared behavior. During seizures, the ACh level in the OFC decreased slowly over the course of several seconds to tens of seconds (Figures 5B and 5C) with a timescale dynamic resembling an awake/sleep transition.^47,53^ Moreover, we found that fast ACh evoked responses at rewarded sound presentation in OFC occurred at a timescale of several hundred milliseconds (Figure 5E) and were impaired during ictal miss responses. The presence of these slow and fast dynamics in OFC supports the hypothesis that there are two modes of cortical cholinergic input: a tonic release (volume transmission) that slowly changes levels of extracellular ACh mediating arousal states, and a phasic release (synaptic) that quickly mediates precisely defined cognitive operations.^53–57^ The altered slow and fast cholinergic neurotransmission we observed in the cortex during focal limbic seizures and the relationships we found to impaired behavior suggest that both slow and fast mechanisms contribute to depressed arousal and impaired cognitive function.

These findings suggest a conceptual framework for understanding the effects of impaired arousal on behavioral responsiveness and conscious experience. In this framework, both slow arousal effects before stimuli as well as fast transient arousal modulation after stimuli contribute synergistically to conscious perception. Slow state-related effects on baseline arousal reduce the overall attentional vigilance level, thereby reducing the probability of perceiving salient sensory stimuli under normal conditions^58–63^ and in disorders of consciousness.^64–68^ Meanwhile, fast dynamic modulation of arousal *after* the occurrence of sensory stimuli has been less studied, but is increasingly recognized as a potent influence on perceptual awareness.^18,69,70^ Indeed, recent work suggests that a transient “pulse” of subcortical arousal immediately after sensory stimuli may play a key role in modulating subsequent processing that distinguishes perceived from not perceived stimuli across sensory modalities.^71–75^ The present finding of fast increases in cortical ACh after perceived auditory stimuli contributes importantly to this emerging concept, where evoked changes in arousal can modulate perception. Our findings that focal limbic seizures reduce cholinergic arousal on both slow and fast timescales and that both effects are related to impaired behavioral responsiveness further support the concept that both slow (state-related) and fast (dynamic evoked) modulation of arousal contribute synergistically to conscious perception.

The origin of the reduced cortical cholinergic neurotransmission in our awake mouse model was not directly investigated in the present experiments. Studies in the rat model showed decreased cholinergic neuronal firing activity in basal forebrain and pedunculopontine tegmental nucleus during focal limbic seizures.^8,16^ Most frontal cortical cholinergic inputs are known to come from the basal forebrain^48–50,76^ and we showed that electrical stimulation in this region can instantly elevate cortical cholinergic signals during light anesthesia (Figures S8B and S8C), supporting direct cortical basal forebrain cholinergic neurotransmission. On the other hand, pedunculopontine tegmental nucleus cholinergic neurons mainly project into the thalamus and the basal forebrain, and thus indirectly modulate cortical arousal, especially via thalamocortical circuits.^77^ Future investigations on both direct and indirect cholinergic pathways to the cortex will be necessary to fully understand how they contribute to impaired consciousness in the awake mouse model.

Beyond the cholinergic system, it is likely that other neurotransmitters and mechanisms also contribute to impaired consciousness in focal limbic seizures, warranting further investigation. For example, both increased activity in direct inhibitory GABAergic pathways as well as decreased activity in indirect excitatory pathways may contribute to depressed subcortical arousal during seizures.^16,20^ Depressed activity in other subcortical arousal neurotransmitter systems, including glutamate, norepinephrine, and serotonin, may also play a role.^19,78,79^

While we showed that impaired responses to auditory cues were associated with reduced fast evoked cortical ACh release, other network mechanisms could contribute to enhance the unresponsive state. For example, the hippocampus modulates formation of contextual memory and learning and can mediate behavioral responses following a cue through connection to the nucleus accumbens,^80–84^ a region crucial for value-based action selection.^85^ In our context, hippocampal seizures could hinder the process of goal-directed behavior (i.e., retrieving of learned reward location and guiding the task-appropriate appetitive behavior). However, this would not explain why some responses to cues remain normal during seizures. One hypothesis could be that other neuronal circuits would compensate for this deficit. For example, beside the cholinergic pathways, neurons from basolateral amygdala that project into the nucleus accumbens are known to promote reward-seeking behavior via dopaminergic neurotransmission.^83,85,86^ How hippocampal seizures affect function in the nucleus accumbens or basolateral amygdala will need further investigation in the context of understanding the impairment of cue-reward contingency mechanisms during focal limbic seizures.

The awake mouse model presented here offers opportunities and advantages to investigate neuronal mechanisms related to arousal and behavior in focal limbic seizures. With genetic tools available in mice (such as optogenetics or genetically encoded calcium indicators/sensor), more possibilities to explore neuronal networks and mechanisms are possible.^87–90^ Head-fixed mice also provide good control of behavioral environment while allowing for simultaneous electrophysiological recordings or imaging that would otherwise be difficult in a freely moving animal.^91,92^ Thus, the current findings demonstrate altered slow and fast cortical cholinergic arousal related to impaired behavior and open paths for a comprehensive understanding of the fundamental mechanisms for loss of consciousness in focal seizures and potentially other brain disorders.

### Limitations of the study

Our acute mouse model can be used to investigate effects of sudden focal limbic seizures and has the advantage of predictable seizure occurrence. However, this model originates from healthy neural circuits, whereas, in human TLE and in chronic TLE animal models, epileptic circuits show neural modifications and circuit rearrangements, which may be important for pathophysiology.^93–97^ Therefore, our findings should be investigated in future work using spontaneous seizure models. In addition, our cortical recordings focused on the OFC, a representative association cortex area shown previously to exhibit slow waves and depressed arousal in human TLE and rodent models.^3–8^ Other association cortex regions would be a logical target for broader study, given that depressed states of consciousness, including anesthesia, sleep, and seizures, impair function in the frontoparietal association cortex more than in primary sensory cortices.^4,5,98–101^ In addition, future studies of primary cortical areas during perception tasks in seizures would also be valuable. Finally, because our experiments were conducted only in female mice, additional work should be done comparing the results in males and females to increase the generalizability of the results.

## RESOURCE AVAILABILITY

### Lead contact

Further information and requests for resources and reagents should be directed to and will be fulfilled by the lead contact, Hal Blumenfeld (hal.blumenfeld@yale.edu).

### Materials availability

This study did not generate new unique reagents.

### Data and code availability

- All data have been deposited at DataDryad.org and are publicly available at https://doi.org/10.5061/dryad.gb5mkkx0r.
- All original code has been deposited at GitHub and is publicly available at https://doi.org/10.5281/zenodo.13991910.
- Any additional information required to reanalyze the data reported in this work paper is available from the lead contact upon request.

## STAR★METHODS

### EXPERIMENTAL MODEL AND STUDY PARTICIPANT DETAILS

#### Mice

All procedures were performed in accordance with approved protocols of Yale University’s Institutional Animal Care and Use Committee. Adult female C57BL/6 mice (Charles River) at 3–6 months of age (weight, 20–28g) were used in these experiments to take advantage of a slower progression in weight gain throughout the experiments, compared to male mice. All mice were group housed on a normal 12h light/dark cycle, then single housed after surgery. They received food and water *ad libitum*, until the day before the start of the behavioral experiments, when they underwent water restriction. 26 animals were used for awake head-fixed behaving seizure induction experiments, 10 out of the 26 animals were used for additional acute multiunit activity (MUA) recordings, 7 animals were used for awake head-fixed behaving seizure induction and fiber photometry experiments and 3 animals were used for lightly anesthetized electrical stimulation and fiber photometry experiments.

### METHOD DETAILS

#### Surgery

##### Surgery and protocol for awake head-fixed behaving seizure experiments

All surgeries were performed under deep anesthesia with Ketamine (90–100 mg/kg) and Xylazine (9–10 mg/kg). A heating pad was used to keep the body temperature constant at 37°C. The mice were placed in a stereotaxic frame and received either pre-operative and post-operative buprenorphine HCl (0.05 mg/kg), or pre-operative Buprenorphine Ethiqa XR (3.25 mg/kg) for general analgesia. Lidocaine (<5 mg/kg) was subcutaneously injected at the incision site. The skin above the skull was cut and removed for skull access. Burr holes were drilled in the skull with an electrical drill (19007–05, Fine Sciences Tools) according to stereotaxic coordinates relative to bregma (Mouse Atlas, Paxinos and Franklin, 2001^102^). Stereotaxic coordinate used for right orbitofrontal cortex (OFC): AP +2.22 mm, ML −1.25 mm, DV −2.75–2.9 mm; for left or right dorsal hippocampus (HC): AP −1.46–2.30 mm, ML ±1.50, DV −1.37–1.40 mm; and left ventral HC: AP −2.70–3.40 mm, ML +3.00 mm, DV −3.50–4.00 mm. Bipolar Teflon coated stainless steel electrodes (outer diameter: 0.150 mm, 8IE36332TWLE, P1 Technologies, Roanoke Va.) were placed in the right OFC and either (1) the left dorsal HC (*n* = 3/26 mice), (2) the left dorsal and ventral HC (*n* = 4/26 mice), or (3) in the left and right dorsal HC (*n* = 19/26 mice). An additional burr hole was drilled at AP −0.10 mm, ML +2.00 mm relative to bregma for a grounding screw (diameter: 1.6 mm, 8IE3639616XE, P1 Technologies). A custom titanium headplate was affixed to the posterior part of the animal’s skull to give space for electrodes implants. Dental cement (Parkell C&B Metabond) was used to secure the electrodes, screw and headplate, and to cover the exposed skull. Mice received Carprofen (5 mg/kg) subcutaneously before waking up and were allowed one week of recovery before starting auditory task training sessions followed by behavioral experiments. All recordings occurred in awake head-fixed animals, running freely on a wheel. Once experiments were completed, animals were euthanized. Brains were then harvested for histological analysis.

##### Surgery and protocol for multiunit activity recording

To record cortical multiunit activity (MUA), a small craniotomy without removing the dura was performed above the left OFC (centered at AP +2.22 mm, ML +1.25 mm relative to bregma) during electrode implantation, ground screw and headplate fixation surgery (previous section). The craniotomy was surrounded by dental cement and temporarily sealed with silicone (Body Double Fast set, Smooth-On). Mice were allowed one-week recovery before starting auditory task training sessions followed by behavioral experiments. Once seizures were successfully induced for at least 2 successive days, MUA recording was performed the following day. The animal was put on the wheel head-fixed and the silicone was gently removed. Sterile saline was used to clean the exposed brain surface and a high-impedance (3–4 MΩ) monopolar tungsten microelectrode (UEWMGGSEDNNM, FHC), was slowly lowered to reach the OFC (SI: −2.00–2.15 mm relative to cortex). Once the target was reached, the usual behavioral experimental session would start. At the end of behavioral experimental session, the microelectrode was slowly removed from the brain and silicone was used to seal the craniotomy. To mark the location of the MUA electrode, two methods were used, either (1) the electrode was dipped into fluorescent DiI staining solution (DiIC_18_, Thermo Fisher Scientific), before lowering it into the brain, or (2) the electrode was gently tapped at the end of a recording session to lightly damage the surrounding brain tissue. If DiI dye was used, the MUA electrode was dipped ten times into the solution, with at least 5 s between each dip. MUA recording was performed for a maximum of 4 behavioral experimental sessions, with one session per day. Then, the mice were euthanized and their brains harvested for histological analysis.

##### Surgery and protocol for fiber photometry experiments

To measure cortical acetylcholine levels, 0.3 μL of AAV9-hsyn-ACh4.3 (ACh3.0) (WZ Biosciences Inc.)^46,47^ was delivered into the left OFC (AP +2.22, ML +1.2, DV −2.75 mm relative to bregma) at a rate of 0.04–0.08 μL/min (Quintessential Stereotaxic Injector, Cat No. 53311, Stoelting Co.). A mono fiber-optic cannula (1.25 mm outer diameter metal ferrule; 2.5 mm long, 400 μm core diameter/430 μm outer diameter, 0.66 numerical aperture (NA), borosilicate; Doric Lenses) was then implanted in left OFC, above the injection site (AP +2.22, ML +1.2, DV −2.6 mm relative to bregma), and fixed to the skull using dental cement (Metabond). Bipolar electrode implantations in OFC and in bilateral HC were also performed along with a ground screw implant and head-plate fixation as already described for awake head-fixed seizures experiments. For basal forebrain electrical stimulation testing during light-anesthesia (Ketamine, 50 mg/kg and Xylazine, 5 mg/kg), additional bipolar electrodes were implanted in bilateral nucleus basalis (NB) (AP −0.6 mm, ML +/−2.0 mm, DV −4.74 mm relative to bregma). A minimum of eight days were allowed for sufficient expression and recovery before start of training, followed by behavioral experiments. Experiments were performed typically two weeks after the injection. Once experiments were completed, animals were euthanized, and brains were harvested for histological analysis.

#### Behavioral experiments: Auditory task and wheel speed recording

Mice were water-restricted beginning one day before the start of training sessions to promote appetite response to liquid reward during behavioral studies and awake recordings. Animals were then acclimated to the experimental set up, including head-fixation and the running wheel, and the auditory task training, which was typically achieved after 2–3 days. They were trained to lick from a lick port containing a drop of water in response to an auditory click stimulus. The auditory stimulus followed by the initiation of a drop of water were generated from a script created via the graphical sequencer editor from Spike2 software (Cambridge Electronic Design Limited). The auditory stimulus consisted of a 12ms arbitrary waveform (white noise pulse at 0–50Hz, approximately 57 dB click sound) created from the graphical sequencer editor and played from a Power 1401 (Cambridge Electronic Design Limited) digital-to-analog output connected to a headphone speaker (Skullcandy In-ear earbuds). 0.07s after the auditory stimulus, a single square digital pulse was initiated from the Power 1401 digital output to open a solenoid electromagnet valve (a14010700ux0271, Uxcell) for 0.05s to deliver an approximately 5 μL water droplet at the lick port. The volume of the droplet was influenced by the duration of the valve opening and by the height of the gravity-delivered water system, and measured by weighing 10 successive droplets before the start of the recording session. Licks were detected by wrapping a wire around the lick port, which was then connected to the input of Power 1401, and by connecting the animal to ground. Tongue contact with the lick port created a DC voltage step reflecting a metal-to-water junction potential which persisted for the duration of the tongue-port contact. This signal was recorded and digitized via Spike2 at a sampling rate of 1kHz. The auditory stimulus occurred at random intervals every 10–15s during the training session. One training session lasted for a minimum 10min or until the animal stopped licking for at least 4 successive drops. Experimental sessions began after the animal reached 95–100% success of licking water after the stimulus, which was typically achieved after 2–3 days of training.

During behavioral experiments, the same Spike2 script was used to automatically generate the auditory stimulus and deliver the water drop. The auditory stimulus followed by water presentation occurred every 10–15 s at random during a baseline period of at least 60s before a seizure was induced. During the seizure or sham period, the auditory stimulus was switched, via another command in the Spike2 script, to a higher rate of every 3–7s to ensure sufficient stimuli were delivered for testing during the ictal period. For the sham period, the higher 3–7s auditory stimulus rate was used for approximately 15–20s, or approximately the same duration as the corresponding seizure if known. During the early and late postictal periods, the stimuli were switched back to 10–15s intervals and continued until the end of session. The behavioral experimental session ended when the animal stopped licking for a minimum of 4 successive drops or when the session reached a maximum of 2 h. Mice underwent behavioral experiments for a maximum of 5 days a week for a maximum duration of 2 months. During those periods, mice were weighed daily and water restricted to reach up to 18% of body weight loss, with free access to food.

During training and behavioral experiments, mouse movement was monitored on a running wheel. The wheel position was recorded with an encoder (MA3-A10–125-B, USDigital) that reported the shaft position over 360° with a voltage signal, which was digitized at a rate of 1 kHz via the CED power 1401.

#### Electrophysiology recordings and seizure induction/electrical stimulation

Local Field Potential (LFP) recordings were performed via chronic-implanted bipolar electrodes in the HC and OFC, using a Microelectrode AC Amplifier (model 1800, A-M Systems) broad-band filtered from 1 Hz to 500Hz (x1000 gain) for HC and from 0.1 Hz to 10 kHz (x1000 gain) for OFC. OFC signal was further filtered with a model 3363 filter (Krohn-Hite) into LFPs (0.1–100 Hz). For MUA recordings, neuronal signals captured by a high-impedance monopolar tungsten microelectrode were broad-band filtered from 0.1 Hz to 10 kHz and amplified (x1000 gain) by a Microelectrode AC Amplifier (model 1800, A-M Systems). A 400 Hz high-pass filter was further applied by a model 3363 filter (Krohn-Hite) to focus on the MUA signal.^6,7,103^ Electrophysiology signals were digitized with a Power 1401 (Cambridge Electronic Design Limited) at a sampling rate of 1 kHz for LFPs and 20kHz for MUA, and recorded using Spike2 software.

One session of recording included both electrophysiology and behavioral experiments (licking and/or running). Seizures were triggered once per session and per day for a maximum of two months. Sham control stimulations without seizures in the HC were also conducted before seizure stimulation or after seizure recovery on the same day of recording, with alternating order of sham or seizure first (Figure S3A).

To induce a seizure, a 2s stimulus train, consisting of a square biphasic (1ms for each phase) pulses at 60 Hz, was delivered via one implanted bipolar electrode in the HC, using an Isolated Pulse Stimulator (model 2100, A-M Systems). The starting current intensity was chosen based on our pilot experiments. In the rat model, a lower current (200–1500μA) intensity can induce a focal limbic seizure (partial seizure), in which fast seizure activity did not propagate to frontal cortex, while a higher current intensity (2 mA) can generate a focal to bilateral tonic-clonic seizure (secondarily generalized seizure), in which fast seizure activity did propagate to the frontal cortex.^6^ In our mouse model, we noticed that a current amplitude at 25 μA was sufficient to trigger focal limbic seizures in HC, while a current >50 μA increased the probability of inducing focal to bilateral tonic-clonic seizures. Typically, current intensity started at 25 μA and increased with an increment of 2–5 μA for the next trial if no seizure was induced. It is worth noting that focal to bilateral tonic-clonic seizures could be triggered at amplitude <50 μA if the stimulating electrode in HC was not located in the CA1 layers, in particular in CA3, dentate gyrus or outside the HC in neocortex (Figure S2). Lowering the current amplitude in the next session could cause a focal rather than a propagated seizure in some cases. As in previous studies of focal limbic seizures,^6,16,26^ any seizures with secondary generalization based on propagation of epileptiform seizure discharges to the neocortex were excluded from the analysis. For the sham control stimulation, we used the same 2s stimulus train, consisting of a square biphasic (1ms for each phase) pulses at 60 Hz, but with a current amplitude of 1μA that did not trigger any seizure activity.

#### Fiber photometry data acquisition

Fluorescent measurement of ACh level was recorded using a Doric Lenses 1-site Fiber Photometry System, which is a standard 405/470 nm system (FPS_1S_405/GFP_400–0.57, Doric Lenses, Inc). The fluorescence of the ACh3.0 sensor was excited by an LED light delivered at a wavelength centered at 470 nm (460–490 nm, GFP-dependent), while another LED light with a wavelength centered at 405nm (400–410 nm, isosbestic point) was used as reference. The excitation LED light was delivered from an integrated fluorescence mini-cube (ilFMC4_IE(400–410)_E(460–490)_F(500–550)_S, Doric Lenses), controlled by a 2-channel LED driver (LEDD_2, Doric Lenses). Each wavelength, 470nm and 405 nm, was modulated by a sinusoidal carrier at 572.205 Hz and 208.616 Hz, respectively,^104^ from the LED driver, then delivered into the brain through a single fiberoptic patchcord (400 μm core, NA 0.57, Doric Lenses) attached to the fiber implant on the animal via a zirconia sleeve (SLEEVE_ZR_1.25-BK, Doric Lenses). Emission light was collected by the same patchcord, back to the mini-cube to be filter at 500–550 nm, then amplified 10x by a fluorescence detection amplifier (Doric Lenses). The signals were then sent into the fiber photometry console (FPC, Doric Lenses) for data acquisition and were recorded using Doric Neuroscience Studio Software at a rate of 12.0 × 10^3^ samples per second. Data sampling rate was then reduced by a factor 10 (decimated) using a moving average with no overlap by the Doric Neuroscience Studio Software before being saved.

#### Histology

Mice were euthanized with an intraperitoneal injection of Euthasol (>450 mg/kg), then perfused transcardially with 0.2% heparinized phosphate buffered saline (PBS) followed by 4% paraformaldehyde (PFA) in PBS. Brains were harvest and post-fixed overnight in 4% PFA in PBS at 4°C. After being wash three times in PBS, blocks of tissue containing the electrodes tracts and regions of interest were dissected and sliced at 60 μm thickness in coronal sections with a Vibratome (Leica Microsystems). For identification of recording sites by electrode tracts alone, slices were mounted on polarized slides, stained with cresyl violet (FC NeuroTechnologies) and coverslipped with Cytoseal 60 (Epredia). For identification of recording sites by electrodes marked with fluorescent DiI staining solution (DiIC_18_), slices were mounted on polarized slides and coverslipped with Fluoromount-G (Thermo Fisher Scientific). For enhancing identification of ACh3.0 GFP expression from virus injection, slices were incubated overnight at 4^o^C with primary antibody rabbit anti-GFP conjunct with Alexa Fluor 488 (A21311, Invitrogen) (1:1000) in PBS-T solution (0.3% Triton X-, PBS) with 5% normal Donkey serum (Jackson ImmunoResearch), then put on a shaker for 2–3h at room temperature. Slices were then washed in PBS five times, mounted and coverslipped with Fluoromount G. Optical fiber placement tracts were large enough to be readily identified on the same slides as the ACh3.0 GFP virus expression. All slides were imaged using a Leica DM6 B Microscope (Leica). Mice were excluded from analysis if the tips of electrodes tracts, fiber tracts or fluorescence injection sites were not identified at the intended site.

### QUANTIFICATION AND STATISTICAL ANALYSIS

#### Data analysis and statistics

Only one seizure was induced in each recording session. Epileptiform seizure activity was determined as in prior human and animal model recordings of HC seizures,^6,38^ based on the presence of approximately 9–12 Hz repetitive spikes or polyspike-and-wave discharges following electrical stimulation in the HC. Seizures were classified as focal if the seizure activity was confined to the HC or focal to bilateral tonic-clonic if seizure activity propagated into the OFC. Only focal seizures were analyzed.

Analyses of OFC electrophysiology, fiberoptic and behavioral data all used epochs based on LFP recordings of HC seizure activity, with analysis epochs defined as follows: (1) “baseline” corresponded to an epoch of 60 s before the start of the electrical stimulation, (2) “ictal” corresponded to the seizure period that began after the electrical stimulation and ended at the maximum duration of seizure activity recorded from the left or right HC LFP electrodes, (3) “early postictal” corresponded to an epoch of 30 s after the end of the defined ictal period, and (4) “late postictal” corresponded to an epoch of 60 s following the early postictal period. Recordings with an ictal period that did not reach at least 5 s duration were not included in the analysis. For specific analysis of the HC seizure activity ipsilateral and contralateral to the side of HC stimulation (Figures 1E–1H and S4), a different end time for the ictal period was defined for the HC LFP signal on each side.

As described in the main text, we classified behavioral responses into two categories, ‘hit’ for spared response, and ‘miss’ for impaired response, based on first lick latency to the auditory stimulus. A ‘hit’ was defined as a first lick that occurred within a 1 s window after the auditory stimulus, and a ‘miss’ as no lick, or a first lick that occurred after the 1s window following the auditory stimulus. We then classified seizures into three types based on behavioral responses during the ictal period: ‘spared seizure’ = only hits to auditory stimuli, ‘impaired seizure’ = only misses, and ‘mixed seizure’ = both hits and misses.

Because there was a variability of seizures induced in the same animal (spared, impaired and/or mixed), we used number of seizures (recording sessions) as the sample size to compare electrophysiological, behavioral or fiberoptic data between periods (baseline vs. ictal, early postictal, late postictal) and between two seizure types (spared vs. impaired). To correlate electrophysiological or fiberoptic signals and behavioral responses (hit and miss), we instead chose to analyze data by number of events (auditory stimuli) because of the variability of licking responses during each period and throughout a recording session. For analysis of hit and miss behavioral responses, only sound presentations with at least an interval of 4 s between two successive sounds were analyzed. If two auditory stimuli were too close, the earlier stimulus was discarded from the behavioral analysis.

##### Electrophysiology

HC LFP signals were processed under Spike2 software by applying a DC removal filter (0.1s time constant) to remove slow DC shifts. For OFC LFP signals, a custom-written MATLAB script was used to filter out slow artifact generated after the electrical stimulation. This script applied a polynomial fit to the first 10s of the OFC LFP signal after the end of the electrical stimulation, and then subtracted it from the raw signal. Recording sessions with large un-removable stimulation artifacts on the LFP signal were discarded from the LFP analysis. To compare LFP data between periods, all LFP data from the remaining sessions were subjected to fast Fourier transformation (FFT) analysis using a custom-written script with the spectrogram function in MATLAB (window size 1000 ms, frequency resolution 1 Hz) to generate time-frequency data of power spectral density (μV^2^/Hz). For each frequency, power spectral density values at each time point were normalized by dividing by the mean power spectral density in the baseline period for that frequency. Mean normalized power spectral density across seizures was then calculated for each defined period (baseline, ictal, early postictal, late postictal) and log transformed to obtain mean normalized power spectral density (dB/Hz). Due to variable seizure duration, we analyzed the first 15s after the ictal onset. Because of their known physiological interest, the LFP delta (1–4 Hz) power and beta (15–30 Hz) power signal changes were averaged during each period (baseline, ictal, early postictal, late postictal) for each recording, and then the means were calculated across all seizures.

To analyze the electrophysiology signals around the hit and miss responses, an epoch of 2 s before and 2 s after the auditory stimulus was used to analyze the LFP data (see Figure 4). Mean normalized power spectral density (described above) was then calculated across each auditory stimulus responses for each defined period (baseline, ictal, early postictal, late postictal) and log transformed to obtain normalized power spectral density (dB/Hz). The LFP delta (1–4 Hz) power signal changes were then averaged during the ictal period for the hit and miss responses for statistical analysis.

To analyze changes in MUA signals, the root-mean-square voltage (V_RMS_) was calculated, which has been validated as a measure of population neuronal firing in previous studies of epilepsy models^6,19,44,45,103^ (Figure S4). V_RMS_ was calculated in consecutive overlapping 1 s time bins at the original sampling rate for each MUA signal. To calculate the time course of mean percent changes for MUA V_RMS_ (see Figure 2E), we then plotted [(V_RMS_ signal – mean V_RMS_ baseline period)/mean V_RMS_ baseline period] X 100%. Due to electrical stimulation artifact, the V_RMS_ signals from −2 s before the stimulus onset to +1 s after the stimulus offset were removed. Only the first 15s after the seizure onset were plotted for the ictal period due to variable seizure end times. For group statistical analyses (see Figure 2F), average MUA V_RMS_ signal changes were calculated for each period, without cutoff for the ictal period, and averaged across all seizures.

To investigate the relationship between cortical MUA and cortical slow waves during up and down states, and between cortical and HC signals during seizures, the OFC MUA, OFC LFP and HC LFP signals were processed using Spike2 software. The OFC MUA V_RMS_ was calculated in consecutive overlapping 0.05 s time bins, then a DC removal filter (0.5 s time constant) was applied to remove slow DC shifts, and the signals were downsampled to 1000Hz to match the LFP sampling frequency. For the OFC and HC LFP signals, a smoothing filter (0.05 s time constant) was used to remove high frequencies and a DC removal filter (0.5 s time constant) was applied. The relationships between the processed signals were then calculated using the Pearson correlation coefficient (r) with MATLAB (see Figure S4 and Table S1).

##### Behavioral analysis

To analyze licking performance, the lick data time courses (mean lick rate) from 2 s before the auditory stimulus to 2 s after the auditory stimulus, with a non-overlapping bin size of 0.1s, were averaged during each period: baseline, ictal, early postictal and late postictal from each seizure. All data were then averaged for each period across all seizures (e.g., see Figure 1I). For the hit or miss responses, the lick data time courses were averaged across each auditory stimulus response for each period (e.g., see Figure 4B). To analyze the mean lick rate before the auditory stimuli for each period, or for hit vs. miss responses, epochs of −2s–0s before the auditory stimuli were averaged for each period for statistical analysis. The maximum lick rate was calculated by choosing the peak of the lick rate within a window time of −0.1 to 1.05 s around the auditory stimuli, then averaged within each period for each seizure. The mean was then calculated by averaging data of each period across all seizures (e.g., see Figure 1J). The delay to first lick was measured within a time window of 3s after the auditory stimuli, beyond which responses were considered as no lick. The time of the first lick measured after each auditory stimulus was averaged for each period within each seizure. The mean was then calculated by averaging data for each period across all seizures (e.g., see Figure 1K).

Wheel speed was calculated from the change in the wheel position signal over time for each period and averaged for each period across seizures (e.g., see Figure 1L). Because mice were freely running during the experiments and often spontaneously had periods of no running, we selected seizures where mice were running at a minimum wheel speed of 10 cm/s during the baseline period for analysis in comparison to the subsequent periods. All data from selected seizures were averaged for each period across seizures to obtain the mean wheel speed.

The same methods to analyze the licking performance and the wheel speed described above were used for the sham control experiments. For analysis, the sham period duration was equivalent to the ictal period duration recorded from the same recording session.

##### Fiber photometry

To analyze ACh3.0 signals data across the whole seizure, data were processed using custom MATLAB (MathWorks) software. For each seizure data were analyzed beginning 60 s before seizure onset and extending to 90 s after seizure offset. To remove artifacts from movement and hemodynamic change, the isosbestic channel signal (405nm) was scaled to the ACh3.0 fluorescence channel (470nm) signal, fitted using linear regression. The scaled isosbestic signal was then subtracted from the ACh3.0 fluorescence signal and this was then divided by the isosbestic signal to obtain DF/F. The DF/F values were then Z-scored for each seizure as follows: (value - mean across the recording)/standard deviation across the recording. We then calculated the timecourse of change in Z-scored DF/F fluorescence divided by the baseline and defined this as “Seizure-related ACh3.0 change.” These values were plotted as: [(ACh3.0 Z-scored DF/F signal – mean ACh3.0 Z-scored DF/F baseline period)/mean ACh3.0 Z-scored DF/F baseline period] for each period: baseline, ictal, early postictal and late postictal, taking the means across all spared seizures and all impaired seizures, with a displaying cutoff at 15s for the ictal period (e.g., see Figure 5C). For statistical analysis, the seizure-related ACh3.0 change was averaged during each period (without cutoff during the ictal period) for each seizure, and then the mean was calculated across all spared/impaired seizures (e.g., see Figure 5D). The sham control analysis was calculated the same way as described above with the sham period duration equivalent to the ictal period duration recorded from the same recording session.

To analyze event (auditory stimulus) related transient ACh3.0 signals, data from 2 s before the auditory stimulus to 2 s after the auditory stimulus were used. The ACh3.0 signal was corrected by subtracting and dividing by the regression fit of the isosbestic signal to obtain DF/F as described above. The data were then re-baselined to correct for slow signal drift by fitting a first order polynomial to the first 2 s (prior to the auditory stimulus) and subtracting the polynomial from the entire 4s signal timecourse. A *Z* score was then calculated as follows: (value – mean of the signal before the auditory stimulus)/standard deviation of the signal prior to the auditory stimulus. These values were defined as the “Event-related ACh3.0 change.” To analyze event-related ACh3.0 changes after the auditory stimuli and to compare the signals between the baseline hit responses, ictal hit responses and ictal miss responses during the ictal period, a mean time course was plotted for each response type across all events (auditory stimuli) during the ictal period (e.g., see Figure 5E). To further analyze the transient signals observed after the auditory stimuli, the event-related ACh3.0 changes were compared between a pre-auditory baseline (−1000ms to 0) and the first transient ACh release phase (P1, 0–250ms after auditory stimulus), then between the pre-auditory baseline and the second transient ACh release phase (P2, 750–1500ms) as described in the main text, by averaging the event-related ACh3.0 signal changes during each phase (pre-auditory, P1 and P2) for each event. The mean was then calculated for each phase across all events. The same analysis was done during the satiated miss responses from recordings without seizure induction (Figure S7).

##### General statistical analyses

Specific details including the exact value of n, what n represents, definition of center, dispersion and precision of measures, and statistical tests used can be found in the results section and the Figure Legends. To detect significant electrophysiological and behavioral level changes in different defined periods, we compared data values in the baseline period with the ictal, early postictal and late postictal periods using ANOVA with Bonferroni-corrected post hoc multiple pairwise comparisons. To analyze independent data sets based on behavioral classification by comparing the means between hit and miss responses or spared and impaired seizures, the nonparametric Mann-Whitney U test (Wilcoxon rank-sum) was used to detect significant changes. The same statistical test was used to detect significant Ach3.0 GFP level changes by contrasting the independent GFP level changes between spared and impaired seizures. The nonparametric Mann-Whitney U test was also used to detect significant ACh3.0 GFP level changes by contrasting the paired pre-auditory stimulus period versus P1 (0–250ms after auditory stimulus) and the paired pre-auditory stimulus period versus P2 (750–1500ms). All data are shown as mean ± standard error mean (S.E.M). All statistical tests were performed using MATLAB software and significance level was set at *p* < 0.05.

## Supplementary Material

1

## Figures and Tables

**Table:** KEY RESOURCES TABLE

REAGENT or RESOURCE	SOURCE	IDENTIFIER
Antibodies		

Primary antibody rabbit anti-GFP conjunct with Alexa Fluor 488	Invitrogen	RRID: AB_221477
Normal Donkey Serum	Jackson ImmunoResearch	RRID: AB_2337258

Bacterial and virus strains		

AAV9-hsyn-ACh4.3 (ACh3.0)	WZ Biosciences Inc.	AAV9-hsyn-ACh4.3

Chemicals, peptides, and recombinant proteins		

Ketamine	Covetrus	SKU 071069
Xylazine	Covetrus	SKU 033198
Buprenorphine HCl	Covetrus	SKU 059122
Buprenorphine Ethiqa XR	Covetrus	SKU 072117
Lidocaine	Covetrus	SKU 002468
Carprofen	Covetrus	SKU 083911
Dil Stain (1,1′-Dioctadecyl-3,3,3′,3′-Tetramethylindocarbocyanine Perchlorate (‘DiI’; DiIC_18_(3))	Thermo Fisher Scientific	D3911
Euthasol	Covetrus	SKU 009444
Cresyl Violet	FC NeuroTechnologies	CAT# PS102–01
Cytoseal™ 60	Epredia	8310–4
Fluoromount-G	Thermo Fisher Scientific	CAT# 00–4958-02

Deposited data		

Source data for figures	This paper	DataDryad: https://doi.org/10.5061/dryad.gb5mkkx0r
Source code for figures	This paper	Zenodo: https://doi.org/10.5281/zenodo.13991910

Experimental models: Organisms/strains		

C57BL/6 mice (strain: 027)	Charles River	RRID:IMSR_CRL:027

Software and algorithms		

Spike2 software	Cambridge Electronic Design Limited; http://www.ced.co.uk/pru.shtml?spk7wglu.htm	RRID:SCR_000903
Doric Neuroscience Studio Software	Doric Lenses; http://doriclenses.com/life-sciences/software/955-doric-neuroscience-studio.html	RRID:SCR_018569
MATLAB software	Mathworks; http://www.mathworks.com/products/matlab/	RRID:SCR_001622

Other		

Burr for micro drill	Fine Sciences Tools	19007–05
Bipolar Teflon coated stainless steel electrodes	P1 Technologies	8IE36332TWXE
Monopolar tungsten microelectrode	FHC	UEWMGGSEDNNM
Quintessential Stereotaxic Injector	Stoelting Co.	Cat No. 53311
Mono fiber-optic cannula	Doric Lenses	MFC_400/430–0.66_2.5mm_MF1.25_FLT
Solenoid electromagnet valve	Uxcell	a14010700ux0271
Encoder (wheel position)	USDigital	MA3-A10–125-B
CED Power 1401	Cambridge Electronic Design Limited	RRID:SCR_016040
Model 1800 2 Channel Microelectrode AC Amplifier	A-M Systems	RRID:SCR_018946
Model 3363 filter	Krohn-Hite	N/A
Model 2100 Isolated Pulse Stimulator	A-M Systems	RRID:SCR_016677
Integrated fluorescence mini-cube	Doric Lenses	ilFMC4_IE(400–410)_E(460–490)_F(500–550)_S
2-channel LED driver	Doric Lenses	LEDD_2
Single fiberoptic patchcord	Doric Lenses	MPF_400/430/1100–0.57_1m_FCM_MF1.25_LAF
Zirconia sleeve	Doric Lenses	SLEEVE_ZR_1.25-BK
Fiber photometry console	Doric Lenses	FPC

## Highlights

- An awake mouse model replicates variable impaired responses seen in human limbic seizures
- Seizures with impaired responses have stronger cortical slow waves resembling deep sleep
- Seizures with impaired responses have larger slow decreases in cortical ACh release
- Impaired individual responses are associated with reduced fast evoked ACh release
