# Supplementary material for: Slow and fast cortical cholinergic arousal is reduced in a mouse model of focal seizures with impaired consciousness
Source: Cell Rep. Author manuscript; Available in PMC 2025 Feb 12. (PMC11817788; doi:10.1016/j.celrep.2024.115012)
Supplement: 1 [file NIHMS2044443-supplement-1.pdf]

Cell Reports, Volume *43*

Supplemental information

Slow and fast cortical cholinergic arousal is reduced in a mouse model of focal seizures with impaired consciousness

Lim-Anna Sieu, Shobhit Singla, Jiayang Liu, Xinyuan Zheng, Abdelrahman Sharafeldin, Ganesh Chandrasekaran, Marcus Valcarce-Aspegren, Ava Niknahad, Ivory Fu, Natnael Doilicho, Abhijeet Gummadavelli, Cian McCafferty, Richard B. Crouse, Quentin Perrenoud, Marina R. Picciotto, Jessica A. Cardin, and Hal Blumenfeld

Supplementary information

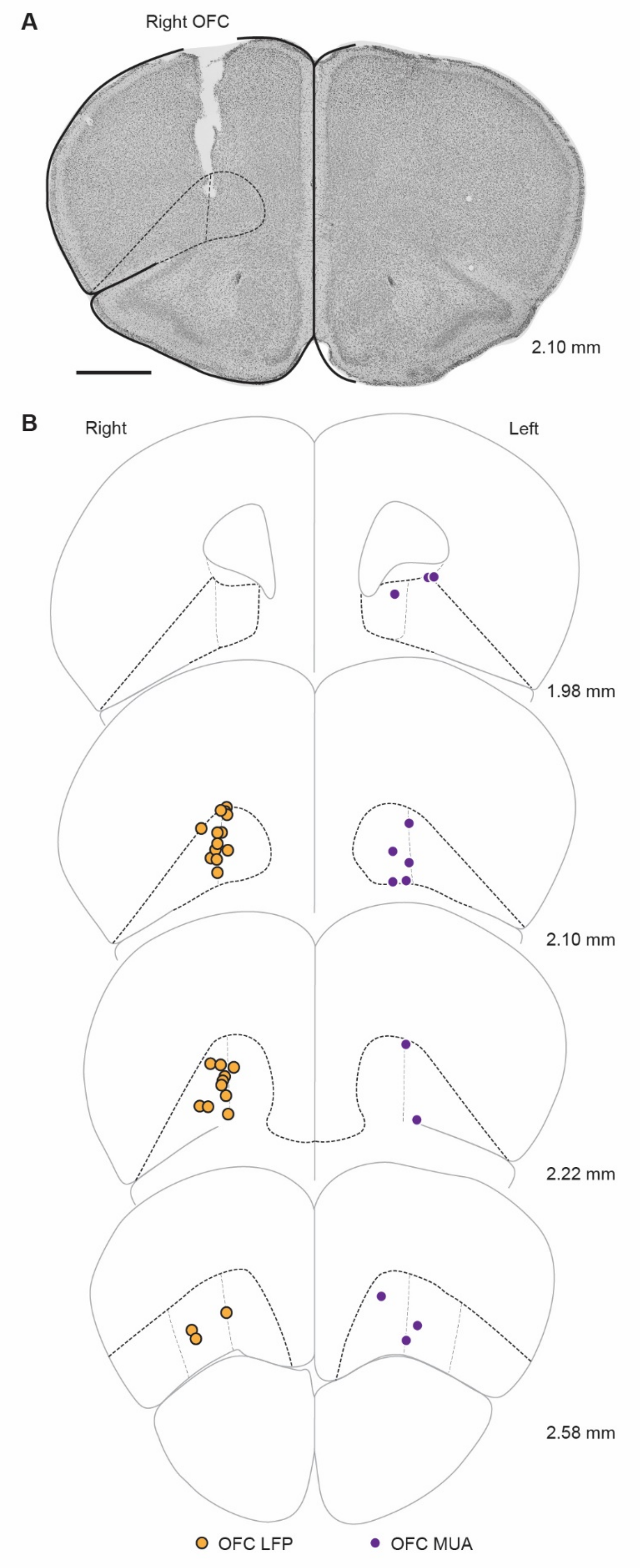

Figure S1. OFC electrode locations, related to Figures 1, 2, 3 and 4. A. Example of brain slices from one animal showing tracts from bipolar LFP electrode in right OFC (black dotted line). Scale bar: 1mm. **B**. Drawing showing every bipolar electrode location used to record right OFC LFP signals (yellow dots) for each animal (n=26) and location of MUA electrodes (purple dots) in left OFC (n=13 recordings in 10 animals, with one animal having 4 different MUA recordings). Adapted with permission from *Mouse Atlas Paxinos & Franklin (2nd ed)* [1].

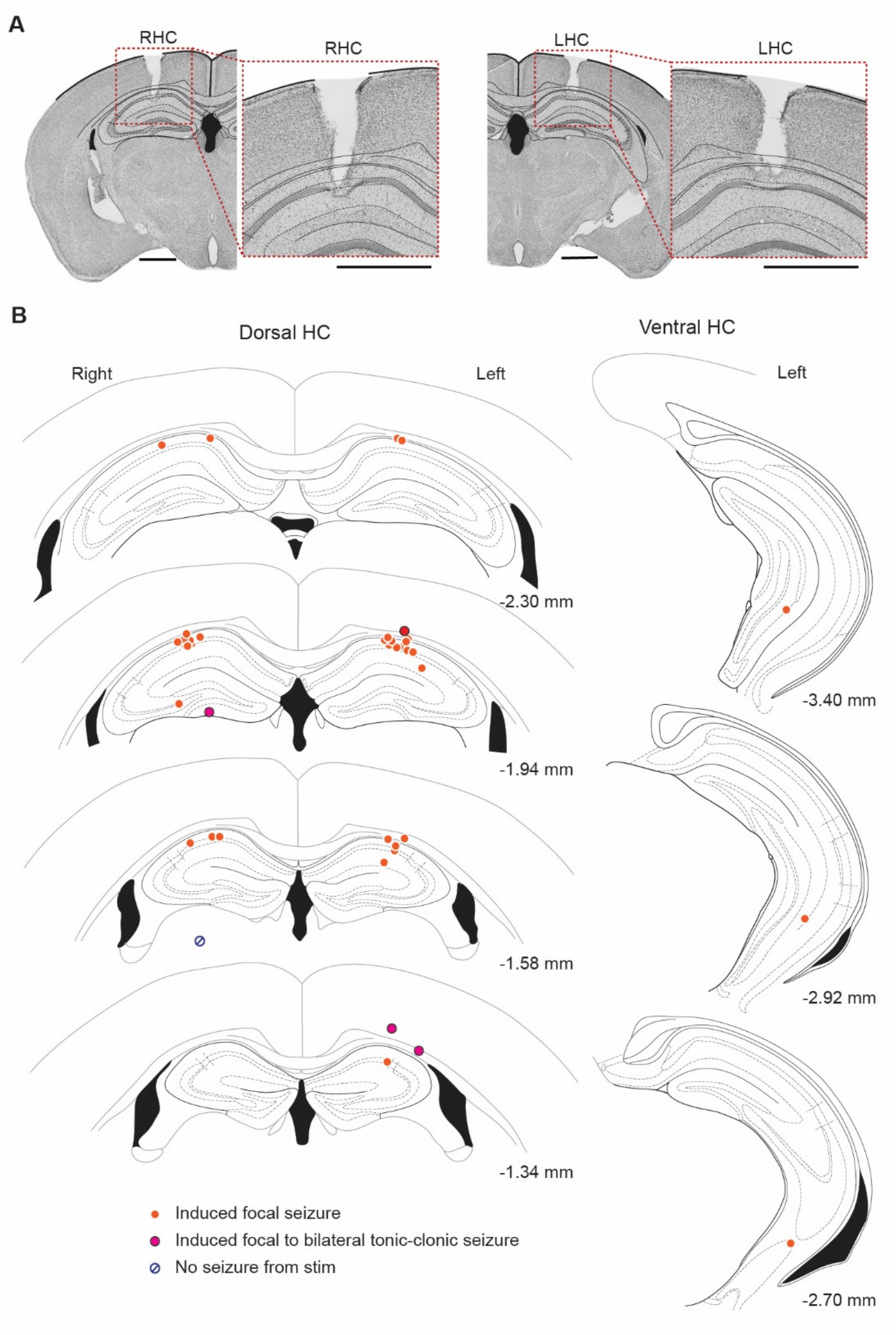

Figure S2. HC electrode locations, related to Figure 1, 2, 3 and 4. A. Example of brain slices from one animal showing tracts from bipolar LFP electrodes in right HC and left HC. Red boxes showed zoom-in electrode trace from right HC (left) and left HC (right). Scale bars: 1mm. **B**. Drawing showing bipolar electrode locations that generate focal

seizures (orange dots, n= 36), focal to bilateral tonic-clonic seizure (pink dots, n= 4) or no seizure (blue crossed out dots, n= 1) in 26 animals. Adapted with permission from *Mouse Atlas Paxinos & Franklin (2nd ed)* [1].

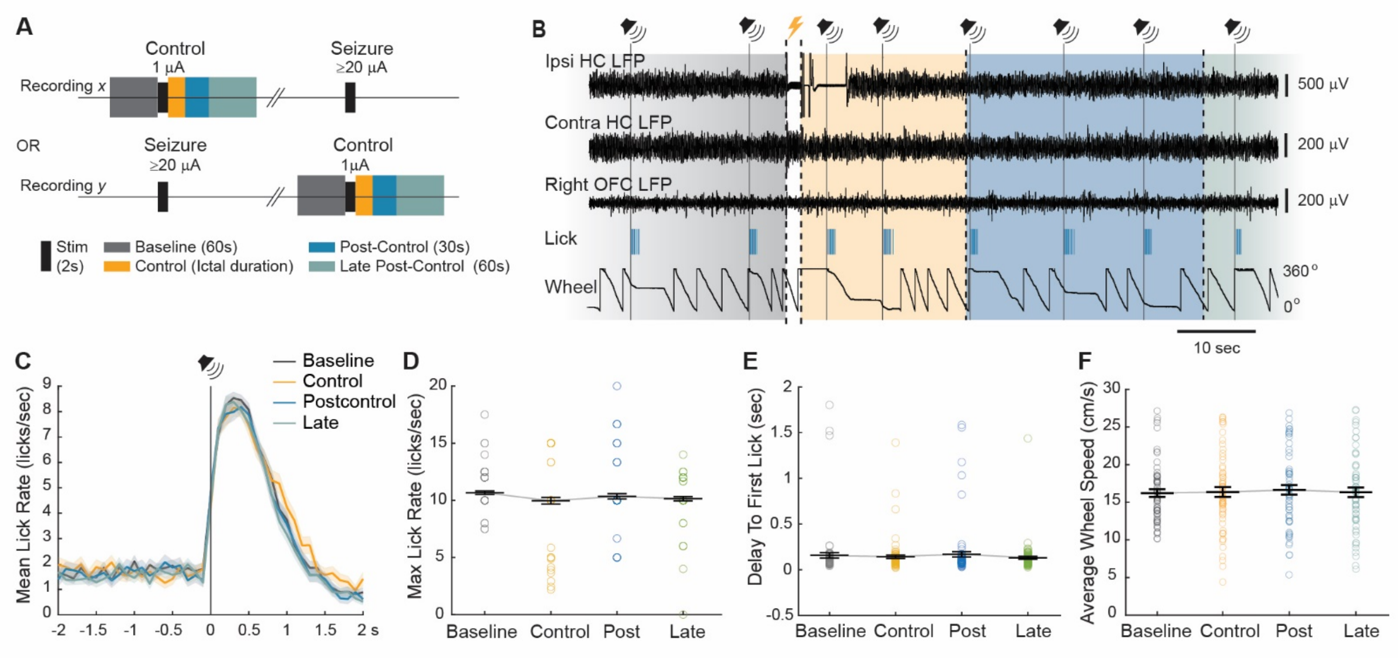

Figure S3. Electrical stimulation without seizure did not impair behavior, related to Figure 1. A. Diagrams illustrating the control electrical stimulation sequence during a recording session. A 2s electrical stimulation that did not induce seizure (Control, 1 µA) was triggered on each day of recording either before or after the triggering of a seizure with a larger stimulus (Seizure, ≥20 µA). Order of seizure first or control first was alternated on each day of recording. Different periods were defined to be comparatively similar to the seizure induction periods of same recording session: Baseline, Control (equivalent to seizure duration), Post-control (equivalent to postictal) and Late post-control (equivalent to late postictal). **B**. Example trace from a control session. The auditory stimuli are represented by vertical lines and speaker symbols. Long vertical dashed lines surrounding the lightning symbol signify the start and end of the artifact from the control electrical stimulus to HC. Shorter vertical dashed lines highlight the end or start of defined periods. No seizure activity is observed in the ipsilateral (Ipsi) and contralateral (Contra) HC local field potential (LFP) following the control electrical stimulation in the Ipsi HC.

Note that the animal continued licking to the auditory stimulus (as indicated by the vertical blue traces), and continued running (indicated by change in wheel position in bottom trace) during the control period after the stimulus. **C**. Mean lick rates aligned to sound presentation (vertical line with speaker symbol) compared at different periods showing similar lick rates. **D-F**. Scatter plot showing average of maximum lick rate (D), average of delay to the first lick (E) following sound presentation for each recording and average of wheel speed (F) during each period for recordings where mice were running (> 10 cm/s) during the baseline period. No significant difference was seen between the different periods. For **C-E**, n=200 control stimuli (recording sessions) in 24 animals. For **F**, n=63 control stimuli with running (> 10 cm/s) during the baseline period in 24 animals. Data are indicated as mean ± S.E.M. Significance was calculated with ANOVA with Bonferroni post-hoc pairwise comparisons of Baseline to each of the other periods with a significance level set at $p<0.05$. Post = post-control period; Late = late post-control period.

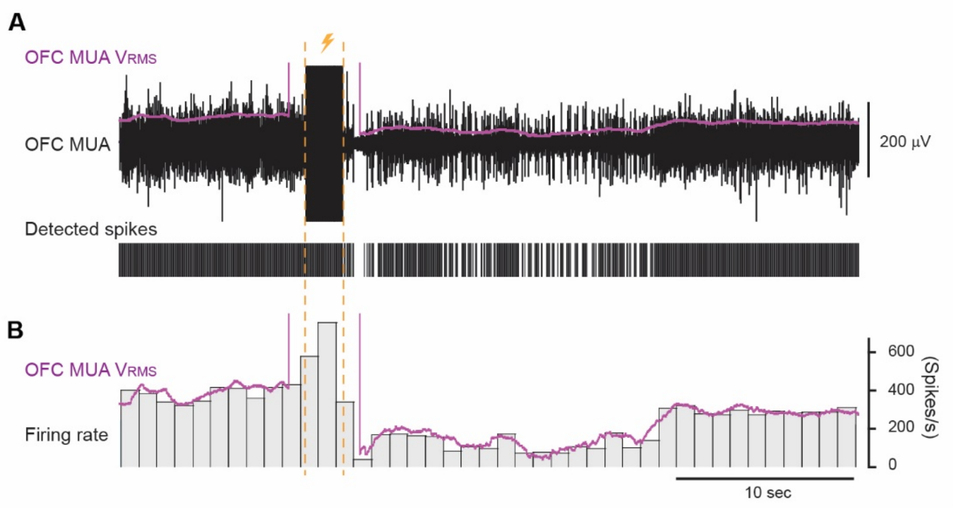

Figure S4. OFC MUA V_{RMS} as surrogate of neuronal firing, related to Figure 2. A. Example of OFC MUA signal (top black trace) with the calculated OFC MUA VRMS (overlayed pink trace), and the detected neuronal MUA spikes using Spike2 (bottom black lines). **B.** Histogram showing the neuronal firing rate (detected spikes/s) and the OFC MUA V_{MRS}. Note that the OFC MUA V_{RMS} closely follow the neuronal firing rate fluctuation.

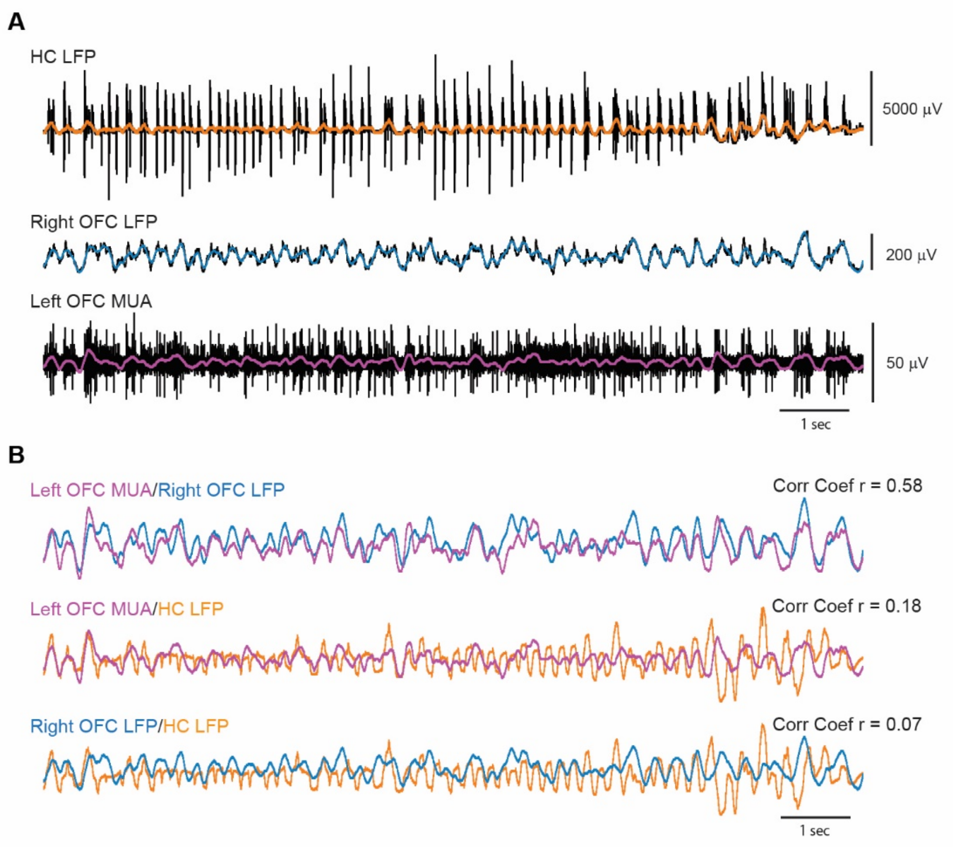

Figure S5. Example of correlations between HC or OFC LFP versus OFC MUA signals during seizure period from one recording session, related to Figure 2 and Table S1. A. Traces showing original signals (black) and processed signals: filtered HC LFP (orange), filtered right OFC LFP (blue) and left OFC MUA V_{RMS} signal (pink). **B.** Comparison between processed signals and the resulting Pearson correlation coefficient r. Top traces represent superimposed left OFC MUA V_{RMS} (pink) and filtered right OFC LFP signals (blue) with a Pearson correlation coefficient r of 0.58. Middle traces represent superimposed left OFC MUA V_{RMS} (pink) and filtered contralateral HC LFP signals (orange) with a Pearson correlation coefficient r of 0.18. Bottom traces represent superimposed filtered right OFC LFP (blue) and filtered contralateral HC LFP signals (orange) with a Pearson correlation coefficient r of 0.07. Note a higher correlation

coefficient between left OFC MUA V_{RMS} and filtered right OFC LFP signals compared to other correlations.

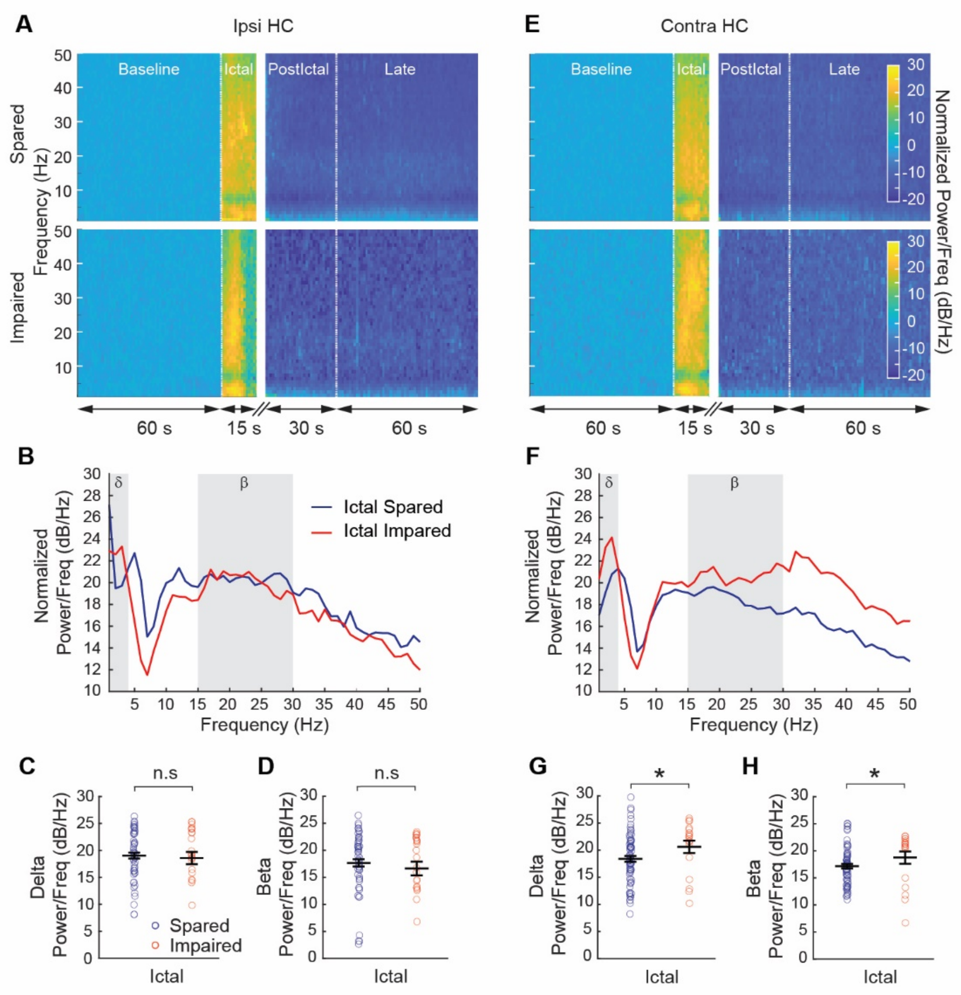

Figure S6. Comparison of spared and impaired seizures in ipsilateral and contralateral hippocampus, related to Figure 3. A. Time-frequency plots showing ipsilateral hippocampus (Ipsi HC) normalized power changes at defined periods: Baseline, Ictal, early postictal (Postictal) and late postictal (Late) periods during spared versus impaired seizures. Normalized Power/Freq (dB/Hz) was calculated by normalizing power spectral density relative to baseline (see Methods). Interestingly, note that large power changes reflecting seizure activity was shorter in the Ipsi HC during the ictal period for impaired seizures. **B**. Power spectrum resulting from the time-frequency plots for the ictal period. Similar power change from the Ipsi HC ictal spared period and ictal impaired

periods are observed. **C**. Mean delta power of Ipsi HC showed no significant difference between spared and impaired seizures. **D**. Mean beta power of Ipsi HC showed no significant difference between spared and impaired seizures. **E**. Time-frequency plots showing contralateral (Contra) HC normalized power changes at defined periods: Baseline, Ictal, early postictal (Postictal) and late postictal (Late) periods during spared versus impaired seizures. **F**. Power spectrum of Contra HC showing power changes during the ictal period. Impaired seizures showed slightly higher power in the delta band and especially in the beta band and higher frequencies during the ictal period. **G**. Mean delta power of Contra HC showed significant increase during impaired seizures compared to spared seizures. **H**. Mean beta power of Contra HC showed significant increase of beta power during impaired seizures compared to spared seizures. For **A-G**, n = 72 spared seizures in 16 animals and 37 impaired seizures in 13 animals, 23 animals in total. Data are indicated as mean ± S.E.M. Significance was calculated with Mann-Whitney U test. *p<0.05 and n.s: non-significant.

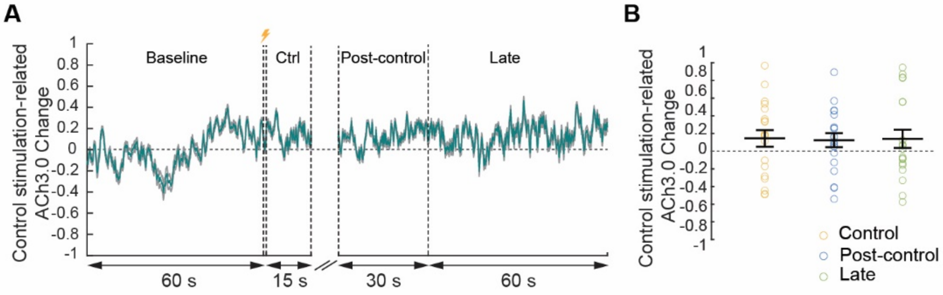

Figure S7. Control sham electrical stimulation without seizure did not elicit ACh3.0 signal changes, related to Figure 5. A. Mean time course of ACh3.0 change from control stimulation recordings (1 µA 60 Hz, 2s train), aligned after the end of the stimulation, then at start of early Post-control period, with analysis performed the same way as during seizures (see Methods). Control stimulation-related ACh3.0 showed no change during the control, early and late post-control periods. Symbols and marking of events during the recording follow the same conventions as in Supplementary Fig S3. **B**. Mean control stimulation-related ACh3.0 changes showing no significant difference between the baseline, control, early and late post-control periods. For **A-B**, n = 20 control stimuli in 5 animals. Data are indicated as mean ± S.E.M. Significance was calculated with Mann-Whitney U test between the Baseline period and each of the other periods with a significance level set at $p<0.05$.

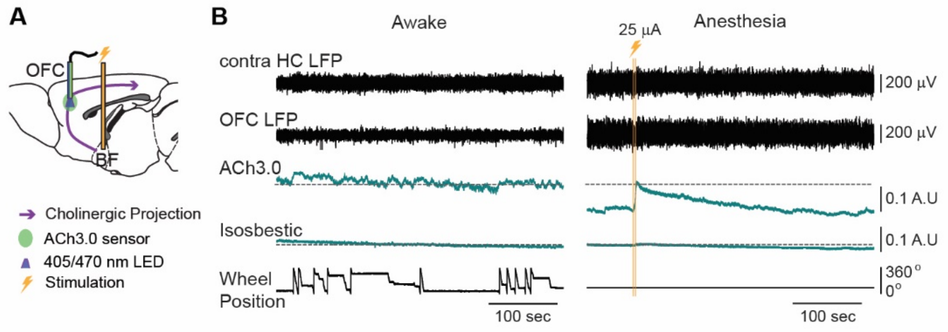

Figure S8. Electrical stimulation in the basal forebrain elicits an increase of Ach3.0 signal during anesthesia, related to Figure 5. A. Diagram illustrating the experimental procedure. Bipolar electrodes were implanted in the left and right nucleus basalis of the basal forebrain (BF), which contains cholinergic neurons that project into the frontal cortex. ACh3.0 sensor was expressed in the left OFC and level of ACh3.0 GFP fluorescence change from the electrical stimulation was measured via an optic fiber. **B**. Example of two recordings from the same animal showing representative signals in the awake state (left) and anesthetized stated (right) with Ketamine/Xylazine 50/5 mg/kg. Note that the level of ACh3.0 signal was higher during the awake state than the anesthetized state (horizontal dashed line indicates baseline awake level). A 3 s bilateral electrical stimulation (lightning symbol) of the BF at 50 Hz (25 μA, biphasic square pulses, 1 ms per phase) during the anesthetized state caused a fast increase of the ACH3.0 signal that briefly reached the level of the awake state but quickly decreased back to anesthetized levels.

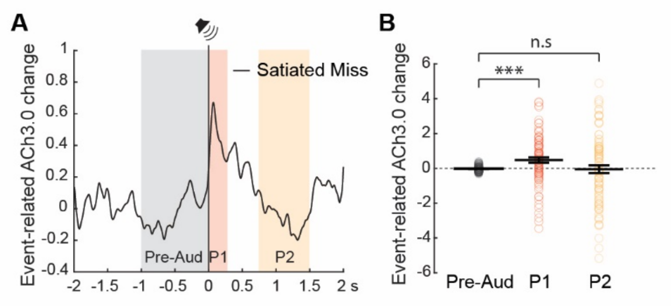

Figure S9. Auditory stimuli after mice became satiated evoked the first ACh release phase but not the second phase, related to Figure 5. A. Mean event-related ACh3.0 change aligned to sound presentation (vertical trace with speaker) during period with consistent miss responses due to satiation, showed the first phase of evoked ACh release (P1, orange box, 0 – 250ms after sound stimulus) but not the second phase of evoked release (P2, yellow box, 750 – 1500 ms). **B**. Mean event-related ACh3.0 changes in the pre-auditory stimuli period (Pre-Aud), the first ACh release phase (P1) and the second release phase (P2) showing significant increases of ACh in P1 versus the pre-auditory period, whereas there was no significant change between the pre-auditory period and P2. For **A-B**, n = 90 stimuli in 27 recordings without seizures, in 7 animals. (mean ± S.E.M.). Data are indicated as mean ± S.E.M. Significance was calculated with Mann-Whitney U test. ***p<0.01 and n.s: non-significant.

Table S1. OFC MUA V_{RMS} are highly correlated with cortical signals, related to Figure 2, see also Figure S5.

Pearson Correlation Coefficient r	OFC MUA V_{RMS} VS OFC LFP	OFC MUA V_{RMS} VS HC LFP	OFC LFP VS HC LFP
Seizure #1	**0.39**	-0.25	0.18
Seizure #2	**0.58**	0.18	0.07
Seizure #3	**0.57**	0.43	0.07
Seizure #4	**0.55**	0.29	0.09
Seizure #5	**0.44**	0.17	-0.19
Seizure #6	**0.49**	-0.06	-0.10
Seizure #7	0.12	0.09	-0.18
Seizure #8	**0.39**	-0.15	-0.36
Seizure #9	**-0.41**	-0.26	0.06
Seizure #10	0.09	-0.14	-0.18
Seizure #11	-0.01	-0.04	-0.02
Seizure #12	**0.63**	0.01	-0.02
Seizure #13	0.38	-0.28	**-0.51**

Notes: Bold values correspond to the highest r value (<-0.3 or >0.3) per seizure recording.

References

1. Paxinos, G., *The mouse brain in stereotaxic coordinates*, K.B.J. Franklin, Editor. 2001, Academic: San Diego, Calif. ;.
